# Safety-Oriented Benchmarking of Large Language Models in Risk-Based Management of Abnormal Cervical Screening Results: Scenario-Based Benchmark Study

**DOI:** 10.2196/98131

**Published:** 2026-09-22

**Authors:** Ömer Osman Eroğlu, Cansın Eroğlu

**Affiliations:** 1 Department of Obstetrics and Gynecology Ankara Etlik City Hospital Ankara Türkiye

**Keywords:** American Society for Colposcopy and Cervical Pathology, ASCCP guidelines, ASCCP, benchmark study, cervical cancer screening, clinical decision support, guideline concordance, large language model, LLM, patient safety, prompt engineering, risk-based management

## Abstract

**Background:**

Large language models (LLMs) are increasingly being considered for clinical decision support, yet their safety in risk-based cervical screening management remains insufficiently characterized.

**Objective:**

This study benchmarked the guideline concordance and safety-related performance of 3 LLMs in the initial American Society for Colposcopy and Cervical Pathology (ASCCP) risk-based management of abnormal cervical screening results, using a purposively constructed synthetic scenario set that oversamples complex and history-dependent decision nodes.

**Methods:**

We developed 60 synthetic clinical scenarios reflecting initial abnormal screening management in immunocompetent, nonpregnant women aged 25 to 65 years using a predefined scenario coverage matrix. GPT-5.3, Gemini 3 Flash, and DeepSeek V3.2 were tested under 2 prompt conditions: Baseline Clinical Prompt and Guideline-Directed Prompt Package. Each scenario was run in 3 independent repetitions per model and prompt arm (1080 total observations). Responses were evaluated by 2 obstetrics and gynecology specialists, blinded to model and prompt-arm identity but not independent of gold-standard construction, using a prespecified rubric. The primary end point was the unsafe major error-free rate. Proportions are reported with CIs adjusted for within-scenario clustering, and generalized estimating equations were used for inferential comparisons.

**Results:**

Under the guideline-directed prompt package, the unsafe major error-free rate was 100% (95% CI 94%-100%) for GPT-5.3, 98.9% (95% CI 94%-99.8%) for Gemini 3 Flash, and 75% (95% CI 63.2%-84%) for DeepSeek V3.2. In the main-effects model, the guideline-directed prompt package was associated with higher odds of both unsafe major error-free performance (odds ratio [OR] 3.76, 95% CI 2.45-5.77; *P*<.001) and exact concordance (OR 8.27, 95% CI 4.14-16.52; *P*<.001). Error rates increased substantially with scenario complexity, rising from 3.1% in low-complexity to 29% in high-complexity scenarios. The most frequent error subtypes were undermanagement, genotype misinterpretation, and history neglect. Interrater agreement was almost perfect (weighted κ=0.839, 95% CI 0.811-0.867).

**Conclusions:**

Safety-oriented benchmark performance in initial ASCCP risk-based management varied markedly by model, prompt condition, and scenario complexity. The guideline-directed prompt package was associated with improvement in both safety and guideline concordance, but even the best-performing model remained vulnerable in complex, history-dependent scenarios. LLMs may have value as clinician-supervised decision support tools, but these benchmark findings should not be interpreted as supporting autonomous clinical use in cervical screening management.

## Introduction

Cervical cancer screening is one of the most successful cancer prevention strategies in women’s health; however, its clinical effectiveness depends not only on the performance of screening tests but also on the accurate and timely management of abnormal results [1]. In this context, the risk-based management consensus guidelines published by the American Society for Colposcopy and Cervical Pathology (ASCCP) in 2019 introduced a major paradigm shift in the management of abnormal cervical screening results [1]. Unlike earlier algorithm-based approaches, these guidelines established a decision framework based on the estimated risk of cervical intraepithelial neoplasia grade 3 or worse (CIN 3+), integrating the current test result with prior screening history, HPV-based testing, genotype information, colposcopic and biopsy findings, and treatment history [1-3]. Six distinct clinical action thresholds were defined, and management decisions are determined according to these thresholds [1]. The guidelines were subsequently revised through formal updates [4] and evolved under the Enduring Consensus Guidelines framework [5], a standing process in which recommendations are revised continuously as new evidence emerges rather than through periodic wholesale revision. The resulting decision environment is therefore dynamic: management thresholds and triage options may change between formal guideline editions. The integration of emerging triage tools, such as dual stain testing, into the same risk-threshold architecture further illustrates that cervical screening management is becoming an increasingly dynamic decision domain [6].

Although the ASCCP risk-based framework offers more precise and individualized clinical management, it has also substantially increased the complexity of the decision-making process. The same current screening result may require entirely different initial management depending on prior human papillomavirus (HPV)–based test results, colposcopy history, or histopathologic background [1-3]. In particular, history-dependent decision nodes, genotype-sensitive distinctions involving HPV 16/18, borderline colposcopy thresholds, and expedited treatment decisions transform this domain from a simple algorithmic application into one requiring contextual clinical reasoning [2,3]. This complexity poses a significant challenge not only for AI systems but also for clinicians. Indeed, the ASCCP Cervical Cancer Screening Task Force has reported difficulties in achieving guideline-concordant practice during the adoption of new screening strategies and noted that the transition period in clinical practice has been longer than anticipated [7].

Large language models (LLMs) have rapidly expanded across diverse health care applications in recent years, including information access, patient education, clinical documentation, and decision support [8]. However, apparently high performance on medical tasks is not equivalent to clinically safe behavior. Contemporary benchmark literature emphasizes that multiple-choice or short-answer tests do not adequately reflect real-world clinical performance and that open-ended, scenario-based, and safety-oriented evaluation frameworks are more informative [9,10]. Wang et al [10] evaluated 6 LLMs within a dual framework of clinical safety and effectiveness and demonstrated that performance declined by an average of 13.3% in high-risk clinical scenarios. Multiple-choice and closed-format evaluations, by contrast, may overestimate readiness for open-ended clinical decision-making, since they constrain the response space and do not require the model to construct a management plan [11]. Gaber et al [9] benchmarked LLM workflows, including a retrieval-augmented configuration, on 2000 intensive care cases and found that the models could suggest likely diagnoses, appropriate specialists, and urgency of care, while continuing to struggle with nuanced clinical data. Ong et al [12] tested an LLM as a medication safety clinical decision support system across 16 clinical specialties and demonstrated that the model improved the accuracy of medication chart review when used alongside pharmacists, compared with either the model or the pharmacist working alone. Together, these findings suggest that LLMs should assume an assistive rather than autonomous role in clinical decision support. Furthermore, recent multirun studies have shown that accuracy and consistency can behave independently when the same inputs are repeatedly presented to the same model and that high accuracy does not necessarily correspond to high stability [13,14].

Only a limited number of studies have evaluated LLM performance in the field of cervical cancer screening and management. Yurtcu et al [15] assessed ChatGPT’s responses to frequently asked questions about cervical cancer and reported acceptable performance at a general knowledge level, while demonstrating that accuracy declined for guideline-based questions. Kuerbanjiang et al [16] tested 9 different LLMs using 100 standardized questions related to cervical cancer management and observed that prompt engineering could improve performance; however, they combined 5 different guidelines and did not use history-dependent scenario design. Angyal et al [17] developed a customized GPT model as a patient education tool in cervical cancer screening and reported favorable usability outcomes, but did not assess clinician-level decision safety. Pavone et al [18] tested ChatGPT 4.0, DeepSeek R1, and Gemini 2.0 against European Society of Gynaecological Oncology (ESGO), European Society for Radiotherapy and Oncology (ESTRO), and European Society of Pathology (ESP) guidelines using 50 questions and reported that all models were inadequate in guideline concordance; however, this study did not include prompt comparison, did not classify error subtypes, and did not employ a safety-centered primary end point.

These prior studies share several common limitations: most used accuracy-focused simple question-and-answer formats, did not apply systematic scenario design based on a single guideline, did not test history-dependent scenarios in which prior screening history alters management, did not classify error profiles from a clinical safety perspective, and did not evaluate the effect of prompt strategy on performance in a controlled manner. To our knowledge, no benchmark study has specifically focused on the 2019 ASCCP risk-based initial management approach, systematically sampled history-dependent decision nodes, employed a clinical safety-centered primary end point, and evaluated the effect of the guideline-directed prompt package in a controlled design.

In the present study, we comparatively evaluated the guideline concordance and safety-oriented benchmark performance of 3 publicly accessible LLMs (GPT-5.3, Gemini 3 Flash, and DeepSeek V3.2) in the initial management of abnormal cervical screening results arising in an immunocompetent, nonpregnant, routine screening population, using 60 predefined synthetic clinical scenarios based on the 2019 ASCCP risk-based management guidelines. The primary end point was the unsafe major error-free rate, and the main secondary end point was the rate of exact concordance with the prespecified gold-standard management decision. The study additionally aimed to examine the effect of a guideline-directed prompt package strategy on model performance, the distribution of errors according to scenario complexity, the clinical patterns of error subtypes, and intramodel consistency.

## Methods

### Ethical Considerations

The study was conducted within the Department of Obstetrics and Gynecology, Ankara Etlik City Hospital. The Clinical Research Ethics Committee of Ankara Etlik City Hospital granted an ethics exemption based on the exemption petition filed by the principal investigator (reference number 308763928; March 18, 2026), on the grounds that the study did not involve real patient data, biological specimens, or human participants. The overall study flow is summarized in Figure 1.

**Figure 1:**
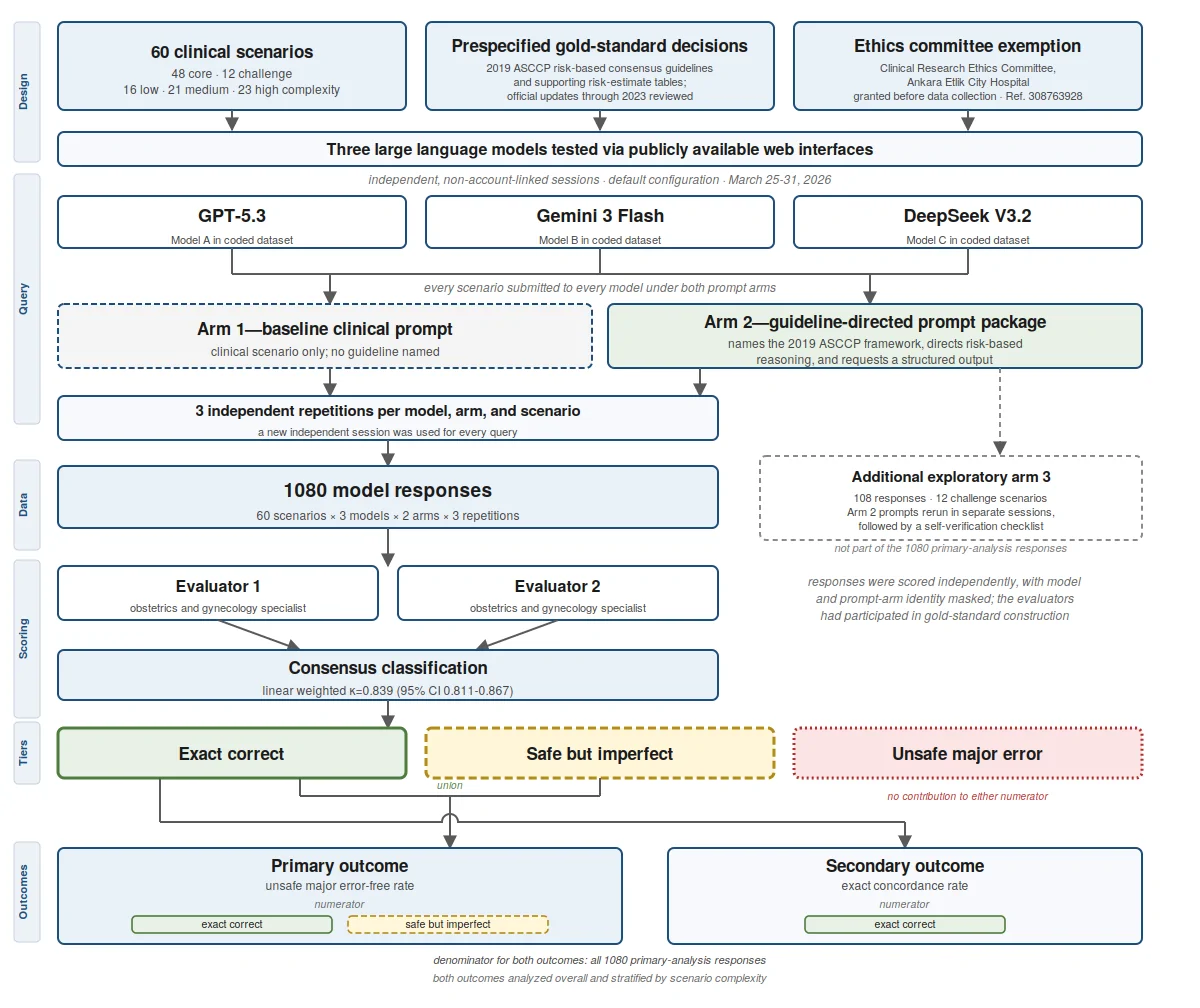
Study flow from scenario construction and ethics exemption through model querying, independent evaluation, 3-tier classification, and outcome derivation. ASCCP: American Society for Colposcopy and Cervical Pathology.

### Study Design

This study was designed as a scenario-based, comparative, observational benchmark study to evaluate the guideline concordance and safety-oriented benchmark performance of LLMs in the initial management of abnormal cervical screening results according to the 2019 ASCCP risk-based approach. No real patient data were used; all inputs consisted of synthetic but clinically realistic scenarios developed on the basis of a predefined scenario coverage matrix. The study design is consistent with the contemporary benchmark literature, which emphasizes that the evaluation of open-ended clinical decision tasks should assess not only accuracy but also safety and consistency as separate dimensions [9,10,12-14].

The study focused exclusively on initial abnormal screening management. Postcolposcopy surveillance, posttreatment follow-up, pregnancy, immunosuppression, HIV infection, transplant recipients, and other special high-risk subpopulations were excluded from the scope. The target clinical framework was the initial management of abnormal cervical screening results arising in an immunocompetent, nonpregnant, routine cervical cancer screening population aged 25 to 65 years [1-6].

### Gold Standard and Guideline Framework

Scenario-level answer keys were anchored to the 2019 ASCCP Risk-Based Management Consensus Guidelines and the supporting studies that established the risk-estimation framework underlying these guidelines [1-3]. Official updates through 2023 were reviewed for currency during post hoc source verification. One scenario concerns the repeat interval for unsatisfactory cytology, for which the prespecified answer key reproduces the 2019 wording; the 2023 update subsequently removed the minimum 2-month interval while retaining the 4-month upper limit. Because the 2023 revision removed the minimum 2-month interval while retaining the 4-month upper limit, it permits earlier rather than later repeat testing. All unsafe major errors recorded for this scenario were classified as undermanagement, which reflects delayed or omitted escalation rather than an interval that was too short; applying the updated wording would therefore not have altered any classification in this dataset. The enduring consensus process and dual-stain recommendations were considered as contextual framework materials and did not alter any answer key. The prespecified decision for each of the 60 scenarios, together with its ASCCP source reference, is provided in Multimedia Appendix 1.

For each scenario, the accepted correct initial management decision was prespecified according to the ASCCP clinical action thresholds based on CIN 3+ risk. Within this framework, the following management options were used as reference: treatment, situations in which either treatment or colposcopy or biopsy was acceptable, colposcopy or biopsy, 1-year surveillance, 3-year surveillance, and return to routine screening at 5 years [1,2].

### Prespecified Evaluation Rubric

To minimize evaluator subjectivity, a scenario-level scoring workbook was prepared before data collection. For each scenario, it specified the scenario code and complexity level, the key clinical inputs, the relevant ASCCP decision node, and the accepted correct initial management decision. Minor and major deviations and their subtypes were assigned by consensus during evaluation using the operational categories available in the scoring workbook; a separate written adjudication manual was not created, and this is reported as a limitation. This structure was designed to distinguish clinically safe but imperfect responses from major errors carrying clinically meaningful harm potential [9,10,12-14]. The accepted correct decision recorded in the workbook is the preferred management option used for exact-concordance scoring. Where the guideline states that 2 options are equally acceptable, both were recorded and both were scored as exact correct. Where the guideline names a preferred option together with an acceptable alternative, only the preferred option was recorded; a response selecting the acceptable alternative was classified as safe but imperfect rather than as an error.

### Development of Clinical Scenarios

A total of 60 synthetic clinical scenarios were developed on the basis of a predefined scenario coverage matrix. Scenarios were not selected at random; rather, they were systematically structured to cover the key decision nodes of ASCCP risk-based initial management. Scenarios were divided into 2 main groups: core scenarios (n=48) and challenge scenarios (n=12).

Core scenarios were designed to systematically sample the major decision nodes of ASCCP risk-based initial management. Scenario development was based on combinations of the following variables: age group, HPV result (negative, positive non–16/18, HPV 16 positive, and HPV 18 positive), cytology result (negative for intraepithelial lesion or malignancy [NILM], atypical squamous cells of undetermined significance [ASC-US], low-grade squamous intraepithelial lesion [LSIL], atypical squamous cells, cannot exclude high-grade squamous intraepithelial lesion [ASC-H], high-grade squamous intraepithelial lesion [HSIL], and atypical glandular cells [AGC]), prior screening history (unknown, negative HPV-based test, and negative cytology alone), and prior biopsy or treatment history. This approach was designed to reflect the ASCCP decision logic, in which the current result is interpreted not in isolation but together with prior history and risk context [1-3].

Challenge scenarios were specifically designed to test situations in which models were expected to encounter difficulty, including scenarios in which the same current result required different management depending on prior history, genotype-specific exception rules involving HPV 16/18, combinations approaching the expedited treatment threshold, age-dependent decision distinctions, and cases in which prior screening history consisted only of cytology-based testing.

The complete list of scenarios is provided in Multimedia Appendix 2.

### Scenario Complexity Classification

Each scenario was assigned a priori to 1 of 3 complexity levels. Low complexity (n=16) represented standard guideline application scenarios in which correct management could largely be determined from the current test result alone. Medium complexity (n=21) represented scenarios requiring integration of at least one prior clinical variable in addition to the current result. High complexity (n=23) represented scenarios involving guideline exceptions, history-dependent management, or multistep decision processes. This classification was used to analyze whether model performance varied not only at an overall level but also according to the difficulty of the clinical decision structure [10,12-14].

### Models Evaluated

Three LLMs were evaluated: GPT-5.3 (OpenAI; released March 3, 2026), Gemini 3 Flash (Google DeepMind; released December 17, 2025), and DeepSeek V3.2 (DeepSeek AI; released December 1, 2025). Final model selection was based on contemporaneous availability during the testing window, free public accessibility through web interfaces, and representation of 3 different model providers. All models were tested using the most current versions accessible during the study period.

Models were accessed through the publicly available web interface of each provider. No API was used. Each query was initiated in an independent, non–account-linked session, so no conversational context was carried over from prior sessions. No explicit web search was invoked, and no response contained source citations indicating retrieval. All models were tested in their free publicly available versions; as no user-configurable reasoning mode was available in the tested interfaces, default settings were used. Scenario development, gold-standard determination, data collection, response evaluation, statistical analysis, and clinical interpretation were performed entirely by the investigators.

### Prompt Design

Each scenario was run in a new and independent session for each model. No prior conversational context, memory, or sequential feedback was carried between sessions. Only the first response was analyzed. The study included 2 main prompt arms.

Arm 1 (baseline clinical prompt): the clinical scenario was presented directly to the model without any guideline name, directive phrasing, or structured output instruction.

Arm 2 (guideline-directed prompt package): The same clinical scenario was presented together with 3 components that were varied jointly rather than separately: the 2019 ASCCP framework was named explicitly, the model was directed to apply the risk-based approach, and a structured output format was requested. Because these 3 components were introduced together, the design cannot separate the contribution of output structure from that of naming the guideline; the arm is therefore described throughout as a multicomponent package.

In addition, an exploratory third arm (arm 3: checklist-based self-verification) was applied to a subset of 12 challenge scenarios. In this arm, the model first generated a response using the arm 2 prompt, after which a structured checklist was presented requesting the model to review and, if necessary, correct its response. This arm was not included in the primary model comparison and was reported solely as exploratory. For arm 3, the arm 2 prompt was rerun in separate independent sessions; these 108 responses were additional to, rather than a subset of, the 1080 primary-analysis responses. The verbatim prompt templates for all 3 arms are provided in Multimedia Appendix 3.

At the end of each prompt, the model was instructed to provide its response in plain text and to conclude with the format “Final Recommendation: [...].” Because the effect of prompt strategy on performance has been reported in prior cervical cancer and general medical LLM studies, the controlled comparison of generic vs guideline-directed prompt package conditions was prespecified in this study [16-18].

### Repetition and Data Collection

Each scenario was run in 3 independent repetitions per model and per prompt arm, thereby enabling intramodel consistency to be evaluated as a separate end point. Each repetition was conducted in a new and independent session. The total number of observations was 1080 for the main analysis (60 scenarios × 3 models × 2 arms × 3 repetitions) and 108 for exploratory arm 3 (12 scenarios × 3 models × 3 repetitions).

Previous medical benchmark studies have demonstrated that accuracy and stability do not always co-occur when identical inputs are repeatedly presented to the same model [13,14]. Accordingly, the triple-repetition approach was defined as a core methodological component of this study.

These parameters—model identity, interface, session independence, and configuration—were fixed and documented at the level of the study protocol. Query-level logs, including build identifiers or response timestamps for individual queries, were not retained. Data collection was conducted between March 25 and March 31, 2026.

### Evaluation Process

Model outputs were evaluated independently by 2 obstetrics and gynecology specialists (ÖOE and CE) in accordance with the prespecified rubric. Responses were presented as coded output sets with model and prompt-arm identifiers removed. The evaluators were therefore blinded to model and prompt-arm identity, but they were not independent of gold-standard construction: both had participated in developing the scenarios and the answer key. Complete masking may also not have been achievable because of the characteristic response styles of different models. These 2 constraints are distinct and are addressed separately in the Strengths and Limitations section. Disagreements between the 2 evaluators were resolved by consensus. An illustrative scoring example demonstrating the 3-tier classification is provided in Multimedia Appendix 4.

### End Points and Error Classification

Each model response was classified into 1 of 3 categories. *Exact correct* was defined as an initial management recommendation fully concordant with the ASCCP guidelines and clinically accurate. *Safe but imperfect* encompassed responses that did not violate the overall framework of clinical safety but contained incomplete phrasing, omission of acceptable alternatives, or minor detail errors. *Unsafe major error* denoted a management recommendation incorrect to a degree carrying clinically meaningful harm potential. This 3-tier classification was based on the principle that not all incorrect responses are clinically equivalent and that a safety-oriented distinction is necessary [10,12,14].

Seven error-subtype options were available in the scoring workbook. Undermanagement was defined as omission or delay of indicated colposcopy or treatment escalation other than an error specific to the expedited-treatment threshold. Overmanagement was defined as recommendation of unnecessary procedures or colposcopy. Wrong expedited treatment was defined as an error specific to the expedited-treatment threshold, that is, recommending expedited treatment where the immediate risk falls below that threshold, or omitting it where the guideline states that expedited treatment is preferred. Wrong surveillance interval was defined as recommending a follow-up interval inconsistent with the guidelines. History neglect was defined as failure to incorporate prior screening history into risk estimation. Genotype misinterpretation was defined as failure to apply exception rules specific to HPV 16/18. Age-threshold error was defined as incorrect application of an age-dependent decision rule. Where an error could in principle satisfy more than 1 definition, the expedited-treatment threshold took precedence: an error was coded as wrong expedited treatment only if it concerned that threshold itself, and as undermanagement otherwise.

The primary end point was the unsafe major error-free rate. The main secondary end point was the rate of exact concordance with the prespecified gold-standard management decision (exact concordance rate). Additional secondary end points included error subtype distribution, performance by scenario complexity, and intramodel consistency. The unsafe major error-free rate was designated as the confirmatory primary end point; all other analyses, although prespecified, were interpreted as exploratory.

### Statistical Analysis

Descriptive proportions are reported with 95% CIs adjusted for within-scenario clustering. The design effect was calculated as 1 + (m − 1) × ICC, where m is the number of observations contributed by each scenario (m=3 for model-by-arm estimates, m=18 for overall complexity-stratum estimates, and m=6 for model-by-complexity estimates) and the intraclass correlation coefficient (ICC) was estimated by 1-way ANOVA separately for each outcome. Wilson intervals were then computed on the effective sample size. In 4 cells (1 model-by-arm cell and 3 model-by-complexity cells), all responses fell in the same category, so the ICC was undefined; for those cells, the number of independent scenarios was used as the effective sample size. A scenario-cluster bootstrap produced closely comparable intervals for the remaining cells. Unadjusted response-level Wilson intervals are provided for comparison in Table S3 in Multimedia Appendix 5.

Between-model comparisons were performed as omnibus and pairwise contrasts from cluster-aware generalized estimating equation (GEE) models, with Bonferroni correction applied across the 3 pairwise comparisons within each outcome and arm. The response-level McNemar test used in the original analysis did not account for the repeated-measures structure and has been removed from the primary inference; the prompt-arm effect is reported from the GEE model, with a complementary scenario-level paired analysis. For that complementary analysis, the 3 repetitions for each scenario, model, and arm were averaged; CIs were obtained by resampling scenarios with replacement, and 2-sided *P* values by exact sign-flip permutation enumeration where computationally feasible, with a Monte Carlo sign-flip test using 200,000 draws where enumeration was not feasible. Between-model heterogeneity in the scenario-level difference was tested by a cluster-robust Wald *F* test from an ordinary least squares model of the difference on model, with SEs clustered by scenario.

GEE logistic regression was used to account for the repeated-measures structure [19]. This approach was chosen to address within-cluster dependence arising from the evaluation of the same scenario across different model, prompt, and repetition combinations. The unsafe major error-free response (binary) and exact concordance (binary) were modeled separately as dependent variables. Fixed effects included model, prompt arm, and scenario complexity; the clustering variable was scenario number. An exchangeable correlation structure was used.

Interrater agreement was calculated using Cohen κ coefficient (unweighted and linear weighted), with CIs for both coefficients derived from the corresponding asymptotic standard error [20]. As a post hoc sensitivity analysis addressing adjudication asymmetry, the primary end point was recomputed separately using each evaluator’s independent ratings. A scenario-cluster bootstrap resampling the 60 scenarios with replacement (4000 replications) yielded 0.801-0.866 for the linearly weighted coefficient, reported alongside the asymptotic interval rather than in place of it. Because several strata contained no unsafe major errors, a Firth penalized logistic regression with profile penalized likelihood confidence intervals was fitted as a sensitivity analysis addressing separation; this model does not account for within-scenario clustering and does not replace the cluster-aware GEEs [21,22]. Intramodel consistency was defined as the proportion of scenarios in which the same consensus classification was obtained across all 3 repetitions.

Arm 3 was analyzed descriptively. Formal significance testing is not reported for this arm because the number of discordant scenarios is too small to support inference.

Statistical analyses were performed using IBM SPSS Statistics (version 26.0; IBM Corp) and Python 3 with the *statsmodels*, *scikit-learn*, and *SciPy* libraries. Analysis code is provided in Multimedia Appendix 6. A *P* value of less than .05 was considered statistically significant.

Sample size was determined by the systematic structure of the predefined scenario coverage matrix. As this study used a scenario-based comparative benchmark design rather than a conventional superiority or equivalence hypothesis test, a precision-based justification approach was adopted instead of a traditional a priori power analysis [23]. With 180 observations per model per prompt arm, the half-width of the CI for proportions between 80% and 100% was projected to remain within approximately ±2.1 to ±5.9 percentage points (pp) before adjustment for within-scenario clustering.

Reporting follows the Chatbot Assessment Reporting Tool (CHART) statement for chatbot health advice studies [24] and the Transparent Reporting of a Multivariable Prediction Model for Individual Prognosis or Diagnosis–Large Language Models (TRIPOD-LLM) reporting guideline for studies using LLMs [25]. A completed CHART checklist is provided in Multimedia Appendix 7.

## Results

### Dataset Characteristics

A total of 1080 model responses (60 scenarios × 3 models × 2 prompt arms × 3 repetitions) were included in the main analysis. This yielded 360 observations per model and 180 observations per model per prompt arm. Of the 60 scenarios, 48 were classified as core scenarios and 12 as challenge scenarios. The complexity distribution comprised 16 low-complexity scenarios (288 observations), 21 medium-complexity scenarios (378 observations), and 23 high-complexity scenarios (414 observations). In addition, 108 observations (12 challenge scenarios × 3 models × 3 repetitions) were collected for exploratory arm 3. The overall characteristics of the study dataset are presented in Table 1.

**Table 1:** Study design and dataset characteristics. Arm 3 was conducted on the 12 challenge scenarios using the arm 2 prompts rerun in separate sessions and is not part of the 1080 primary-analysis responses. The evaluators were masked to model and prompt-arm identity but had participated in construction of the gold standard, as described in the Strengths and Limitations section.

Characteristic		Value, n
Total observations (primary analysis)		1080
Models evaluated		3 (GPT-5.3, Gemini 3 Flash, and DeepSeek V3.2)
Prompt arms		2 (baseline clinical prompt; guideline-directed prompt package)
Total scenarios (n=60)		
	Core scenarios	48 (864 observations)
	Challenge scenarios	12 (216 observations)
Repetitions per scenario (model and arm)		3
Observations per model		360
Observations per model per arm		180
Scenario complexity (low)		16 scenarios (288 observations)
Scenario complexity (medium)		21 scenarios (378 observations)
Scenario complexity (high)		23 scenarios (414 observations)
Additional exploratory arm 3 observations		108
Gold standard		Prespecified gold-standard decisions based on the 2019 ASCCP^a^ risk-based management consensus guidelines and supporting risk-estimate tables; post hoc source verification also reviewed official updates through 2023
Evaluation		Two obstetrics and gynecology specialists, scoring independently with model and prompt-arm identity masked
Primary outcome		Unsafe major error-free rate
Main secondary outcome		Exact concordance rate

^a^ASCCP: American Society for Colposcopy and Cervical Pathology.

### Primary End Point: Unsafe Major Error-Free Rate

Unsafe major error-free rates by model and prompt arm are presented in Table 2. GPT-5.3 produced an unsafe major error-free response rate of 95.6% (95% CI 87.6%-98.5%) in the baseline clinical prompt arm (arm 1), which increased to 100% (95% CI 94%-100%) in the guideline-directed prompt package arm (arm 2). The corresponding rates for Gemini 3 Flash were 80% (95% CI 69%-87.8%) and 98.9% (95% CI 94%-99.8%), respectively, whereas those for DeepSeek V3.2 were 58.9% (95% CI 46.9%-69.9%) and 75% (95% CI 63.2%-84%), respectively. All CIs are adjusted for within-scenario clustering; unadjusted response-level CIs are provided in Table S3 in Multimedia Appendix 5.

In arm 1, the difference among the 3 models was statistically significant in a cluster-aware omnibus contrast (Wald *χ*^2^_2_=26.83; *P*<.001). All 3 pairwise comparisons remained significant after Bonferroni correction (Table S4 in Multimedia Appendix 5). In arm 2, GPT-5.3 produced no unsafe major errors, so the omnibus contrast and the 2 pairwise comparisons involving GPT-5.3 were not estimable for this outcome; the observed rates were 100%, 98.9%, and 75% for GPT-5.3, Gemini 3 Flash, and DeepSeek V3.2, respectively, and the contrast between Gemini 3 Flash and DeepSeek V3.2 remained significant (*P*=.001). The distribution of response classifications across all scenarios by model and prompt arm is visualized in Multimedia Appendix 8.

**Table 2:** Response classification and performance by model and prompt arm. Each model-arm cell comprises 180 responses (60 scenarios × 3 repetitions). Arm 1 is the baseline clinical prompt; arm 2 is the guideline-directed prompt package. CIs are adjusted for within-scenario clustering using a design-effect correction: design effect = 1 + (m − 1) × ICC, m=3, with Wilson intervals computed on the effective sample size. In the GPT-5.3 arm 2 cell, all responses fell in the same category, so the ICC^a^ was undefined and the number of independent scenarios (n=60) was used as the effective sample size. Unadjusted response-level Wilson intervals are provided in Table S3 in Multimedia Appendix 5.

Model	Arm	Exact correct, n	Safe but imperfect, n	Unsafe major error, n	Unsafe major error-free rate, % (95% CI)	Exact concordance rate, % (95% CI)
GPT-5.3	1	156	16	8	95.6 (87.6-98.5)	86.7 (75.8-93.1)
GPT-5.3	2	177	3	0	100 (94-100)	98.3 (95.2-99.4)
Gemini 3 Flash	1	104	40	36	80 (69-87.8)	57.8 (45.3-69.4)
Gemini 3 Flash	2	169	9	2	98.9 (94-99.8)	93.9 (85.6-97.5)
DeepSeek V3.2	1	68	38	74	58.9 (46.9-69.9)	37.8 (26.7-50.3)
DeepSeek V3.2	2	98	37	45	75 (63.2-84)	54.4 (42-66.3)

^a^ICC: intraclass correlation coefficient.

### Performance by Prompt Condition

The guideline-directed prompt package (arm 2) was associated with a higher unsafe major error-free rate in all 3 models compared with the baseline clinical prompt (arm 1). In the GEE model, the odds of an unsafe major error-free response were 3.76-fold higher in arm 2 (95% CI 2.45-5.77; *P*<.001). In a complementary scenario-level analysis, the absolute improvement was +4.4 pp for GPT-5.3 (95% CI 0.0-10.0 pp; *P*=.25), +18.9 pp for Gemini 3 Flash (95% CI 10.0-28.3 pp; *P*<.001), and +16.1 pp for DeepSeek V3.2 (95% CI 7.2-26.7 pp; *P*=.003). Improvement was observed in 3, 14, and 13 scenarios, respectively, with 4 scenarios showing deterioration for DeepSeek V3.2 and none for the other 2 models. For GPT-5.3, only 3 scenarios differed between arms, so the smallest attainable permutation *P* value is .25 and the reported value lies at that floor.

### Exact Concordance With ASCCP Recommendations

Regarding the main secondary end point, GPT-5.3 produced responses fully concordant with the ASCCP guidelines in 86.7% of cases (95% CI 75.8%-93.1%) in arm 1 and 98.3% of cases (95% CI 95.2%-99.4%) in arm 2. The corresponding rates for Gemini 3 Flash were 57.8% (95% CI 45.3%-69.4%) and 93.9% (95% CI 85.6%-97.5%), respectively, whereas those for DeepSeek V3.2 were 37.8% (95% CI 26.7%-50.3%) and 54.4% (95% CI 42%-66.3%), respectively. In both arms, the overall difference among the 3 models was statistically significant in cluster-aware omnibus contrasts (arm 1: Wald *χ*^2^_2_=38.31; *P*<.001 and arm 2: Wald *χ*^2^_2_=44.83; *P*<.001). In arm 2, the pairwise contrast between GPT-5.3 and Gemini 3 Flash did not meet the Bonferroni-corrected threshold (*P*=.04; threshold=.0167).

### GEE Logistic Regression Analysis

The results of the GEE logistic regression analysis, performed to account for the repeated-measures structure, are presented in Table 3. For the unsafe major error-free rate, taking GPT-5.3 as the reference, Gemini 3 Flash showed a significantly lower likelihood of producing a safe response (odds ratio [OR] 0.27; 95% CI 0.16-0.45; *P*<.001). This likelihood decreased even more markedly for DeepSeek V3.2 (OR 0.05; 95% CI 0.02-0.12; *P*<.001). The guideline-directed prompt package increased the likelihood of an unsafe major error-free response approximately 3.8-fold (OR 3.76; 95% CI 2.45-5.77; *P*<.001). In high-complexity scenarios, the likelihood of an unsafe major error-free response was significantly lower than in low-complexity scenarios (OR 0.05; 95% CI 0.01-0.24; *P*<.001), whereas the difference for medium complexity did not reach statistical significance (OR 0.30; 95% CI 0.07-1.37; *P*=.12).

A similar pattern was observed for exact concordance, although effect sizes were more pronounced. The guideline-directed prompt package increased the likelihood of exact concordance 8.3-fold (OR 8.27; 95% CI 4.14-16.52; *P*<.001). Compared with GPT-5.3, DeepSeek V3.2 had a substantially lower likelihood of exact concordance (OR 0.014; 95% CI 0.004-0.045; *P*<.001). High complexity was associated with a marked reduction in the likelihood of exact concordance compared with low complexity (OR 0.005; 95% CI 0.001-0.032; *P*<.001). A model-by-prompt interaction was examined for exact concordance and was not statistically significant on the odds scale (DeepSeek V3.2 × arm 2: OR 0.33, 95% CI 0.08-1.42; *P*=.14 and Gemini 3 Flash × arm 2: OR 2.52, 95% CI 0.47-13.48; *P*=.28). The corresponding interaction model for the unsafe major error-free outcome was not estimable because 9 of the 18 model-by-arm-by-complexity cells contained 0 unsafe major errors. A Firth penalized sensitivity analysis confirmed that the direction of the prompt-package effect was unchanged, whereas the very wide profile penalized likelihood intervals confirmed substantial effect-size instability associated with sparse and 0-event cells (Table S5 in Multimedia Appendix 5). In the complementary scenario-level analysis, between-model heterogeneity in the absolute improvement was statistically significant for both outcomes (unsafe major error-free: *F*_2,59_=6.97; *P*=.002 and exact concordance: *F*_2,59_=6.38; *P*=.003).

**Table 3:** GEE^a^ models for the primary and main secondary outcomes. Binomial GEEs with a logit link, scenario as the clustering unit, an exchangeable working correlation structure, and robust SEs. Both primary main-effects models converged without warnings. Estimates involving strata with no observed unsafe major errors should be interpreted with caution. Nine of the 18 model-by-arm-by-complexity cells contained no unsafe major errors: GPT-5.3 in the low- and medium-complexity strata of arm 1 and in all 3 strata of arm 2, Gemini 3 Flash in the low-complexity stratum of arm 1 and in the low- and medium-complexity strata of arm 2, and DeepSeek V3.2 in the low-complexity stratum of arm 2. A model-by-arm interaction was examined for exact concordance and was not statistically significant (Table S5 in Multimedia Appendix 5); the corresponding interaction model for the unsafe major error-free outcome was not estimable because 9 of 18 model-by-arm-by-complexity cells contained 0 unsafe major errors.

Outcome and term		OR^b^ (95% CI)	*P* value
Unsafe major error-free			
	Gemini 3 Flash vs GPT-5.3	0.269 (0.162-0.447)	<.001
	DeepSeek V3.2 vs GPT-5.3	0.051 (0.022-0.119)	<.001
	Arm 2 vs arm 1	3.756 (2.446-5.767)	<.001
	Medium vs low complexity	0.303 (0.067-1.369)	.12
	High vs low complexity	0.047 (0.009-0.242)	<.001
Exact concordance			
	Gemini 3 Flash vs GPT-5.3	0.145 (0.073-0.289)	<.001
	DeepSeek V3.2 vs GPT-5.3	0.014 (0.004-0.045)	<.001
	Arm 2 vs arm 1	8.269 (4.140-16.518)	<.001
	Medium vs low complexity	0.056 (0.010-0.329)	.001
	High vs low complexity	0.005 (0.001-0.032)	<.001

^a^GEE: generalized estimating equation.

^b^OR: odds ratio.

### Performance by Scenario Complexity

The unsafe major error rate increased markedly with increasing scenario complexity (Table 4). The unsafe major error rate was 3.1% (95% CI 1.1%-8.3%) in low-complexity scenarios, 9.5% (95% CI 5.3%-16.5%) in medium-complexity scenarios, and 29% (95% CI 19.9%-40.1%) in high-complexity scenarios. The corresponding gradient is reflected in the GEE model, in which high complexity was associated with markedly lower odds of an unsafe major error-free response compared with low complexity (Table 3).

When examined by model (Table 5), the most striking findings emerged in the high-complexity group. In high-complexity scenarios, GPT-5.3 produced unsafe major error-free responses in 94.2% of cases, whereas the corresponding rates were 76.8% for Gemini 3 Flash and only 42% for DeepSeek V3.2. In low-complexity scenarios, by contrast, both GPT-5.3 and Gemini 3 Flash achieved a 100% unsafe major error-free rate, whereas DeepSeek V3.2 still produced unsafe major errors in 9.4% of cases even in this group (Table 5).

**Table 4:** Response classification and performance by scenario complexity. Complexity strata pool observations across models and prompt arms, so each scenario contributes 18 observations (3 models × 2 arms × 3 repetitions); the design-effect correction therefore uses m=18 for these estimates, and ICCs^a^ were estimated separately for each outcome. The corresponding unsafe major error rates were 3.1% (95% CI 1.1%-8.3%) for low complexity, 9.5% (95% CI 5.3%-16.5%) for medium complexity, and 29% (95% CI 19.9%-40.1%) for high complexity.

Complexity	Observations, n	Exact correct, n	Safe but imperfect, n	Unsafe major error, n	Unsafe major error-free rate, % (95% CI)	Exact concordance rate, % (95% CI)
Low	288	275	4	9	96.9 (91.7-98.9)	95.5 (90.1-98)
Medium	378	297	45	36	90.5 (83.5-94.7)	78.6 (69.1-85.8)
High	414	200	94	120	71 (59.9-80.1)	48.3 (39.5-57.3)

^a^ICC: intraclass correlation coefficient.

**Table 5:** Performance by model and complexity stratum. Each scenario contributes 6 observations (2 arms × 3 repetitions), so m=6 for those estimates. In 3 cells, all responses fell in the same category and the ICC^a^ was undefined; for those cells, the number of independent scenarios was used as the effective sample size, as in Table 2. This panel is descriptive and was not used for formal inference.

Model and complexity			Observations, n		Unsafe major errors, n (%)	Unsafe major error-free rate, % (95% CI)
GPT-5.3						
	Low	96		0 (0)		100 (80.6-100)
	Medium	126		0 (0)		100 (84.5-100)
	High	138		8 (5.8)		94.2 (84.4-98)
Gemini 3 Flash						
	Low	96		0 (0)		100 (80.6-100)
	Medium	126		6 (4.8)		95.2 (86.3-98.5)
	High	138		32 (23.2)		76.8 (64.7-85.7)
DeepSeek V3.2						
	Low	96		9 (9.4)		90.6 (76.5-96.6)
	Medium	126		30 (23.8)		76.2 (61.7-86.4)
	High	138		80 (58)		42 (25.8-60.2)

^a^ICC: intraclass correlation coefficient.

### Unsafe Major Error Subtypes

A total of 165 unsafe major errors were identified. The most frequently observed error subtype was undermanagement, accounting for 39.4% (n=65) of all errors. This was followed by genotype misinterpretation (n=48, 29.1%), history neglect (n=37, 22.4%), and overmanagement (n=15, 9.1%; Table 6).

The distribution of error subtypes differed markedly across models. All 8 errors produced by GPT-5.3 were classified as history neglect; this model did not produce any genotype misinterpretation, undermanagement, or overmanagement errors. Among the 38 errors generated by Gemini 3 Flash, undermanagement (n=14), genotype misinterpretation (n=13), and history neglect (n=11) were observed at similar frequencies. DeepSeek V3.2 had the highest overall error burden with 119 errors, and its error profile was dominated by undermanagement (n=51), followed by genotype misinterpretation (n=35), history neglect (n=18), and overmanagement (n=15). Overmanagement errors were observed exclusively in DeepSeek V3.2.

When evaluated by prompt arm, 71.5% (n=118) of all errors occurred in arm 1 and 28.5% (n=47) in arm 2, supporting the conclusion that the guideline-directed prompt package reduced the overall error burden (Table 6).

**Table 6:** Distribution of unsafe major error subtypes. Seven error-subtype options were available in the scoring workbook. Each unsafe major error was assigned 1 primary subtype by consensus of the 2 evaluators. Wrong surveillance interval, age-threshold error, and wrong expedited treatment were available as options but received no consensus assignments. Because subtype assignment was made by consensus without an independent reliability assessment, these zero counts should not be interpreted as evidence that the corresponding error types cannot occur.

Error subtype	Total, n (%)	GPT-5.3, n	Gemini 3 Flash, n	DeepSeek V3.2, n	Arm 1/arm 2, n
Undermanagement	65 (39.4)	0	14	51	48/17
Genotype misinterpretation	48 (29.1)	0	13	35	29/19
History neglect	37 (22.4)	8	11	18	26/11
Overmanagement	15 (9.1)	0	0	15	15/0
Wrong surveillance interval	0 (0)	0	0	0	0/0
Age-threshold error	0 (0)	0	0	0	0/0
Wrong expedited treatment	0 (0)	0	0	0	0/0
Total	165 (100)	8	38	119	118/47

### Interrater Agreement

The raw agreement rate between the 2 evaluators, who scored responses independently, was 88.9% (960/1080). Cohen κ coefficient was 0.770 (95% CI 0.732-0.807) in the unweighted analysis and 0.839 (95% CI 0.811-0.867) in the linearly weighted analysis, corresponding to substantial and almost perfect agreement, respectively, under the benchmarks of Landis and Koch [20] (Table S1 in Multimedia Appendix 5).

Most disagreements occurred between the exact correct and safe but imperfect categories. A total of 81 responses were classified as exact correct by the first evaluator and safe but imperfect by the second, whereas 11 responses showed the opposite pattern. Cross-category disagreements between safe but imperfect and unsafe major error were less frequent, with 13 responses classified in the former-to-latter direction and 15 in the latter-to-former direction.

### Intramodel Consistency

Intramodel consistency, defined as the proportion of scenarios in which the same consensus classification was obtained across all 3 repetitions, varied across models and prompt arms. GPT-5.3 demonstrated complete consistency in 98.3% (59/60) of scenarios in arm 1 and in 95% (57/60) of scenarios in arm 2. The corresponding rates for Gemini 3 Flash were 90% (54/60) and 95% (57/60), respectively, whereas those for DeepSeek V3.2 were 86.7% (52/60) and 93.3% (56/60), respectively. No model-prompt arm combination produced any scenario in which all 3 repetitions received 3 different classifications (Multimedia Appendix 9).

### Exploratory Arm 3: Checklist-Based Self-Verification

A total of 108 observations were evaluated under exploratory arm 3 (Table S2 in Multimedia Appendix 5). Because GPT-5.3 had already produced no unsafe major errors in arm 2, no self-correction was observed. For Gemini 3 Flash, postchecklist correction was identified in 8.3% (3/36) of responses, and for DeepSeek V3.2 in 16.7% (6/36) of responses. Formal significance testing is not reported for this arm because the number of discordant scenarios is too small to support inference. No model showed a reverse transition in which a previously safe response became classified as an unsafe major error after checklist-based review.

## Discussion

### Principal Results

In this scenario-based benchmark study, the performance of LLMs in the 2019 ASCCP risk-based initial management framework was evaluated not only in terms of accuracy but also from the perspective of clinical safety. The principal results of the study can be summarized under 4 main themes. First, a clear performance hierarchy was observed among the 3 models, with GPT-5.3 demonstrating the strongest performance in terms of both the unsafe major error-free rate and exact guideline concordance. Second, the guideline-directed prompt package was associated with improved performance across all models, with larger absolute gains in the models with weaker baseline performance, although the formal interaction test was not statistically significant on the odds scale. Third, the unsafe major error rate increased markedly with increasing scenario complexity, and this deterioration became especially striking at history-dependent decision nodes. Fourth, unsafe major errors were not randomly distributed but clustered around undermanagement, genotype misinterpretation, and history neglect. This pattern suggests that LLMs in this domain are challenged not only by knowledge limitations but also by difficulty in integrating risk context.

One of the most important contributions of this study is that it moved beyond the conventional “correct/incorrect” dichotomy by evaluating performance using a safety-centered primary end point, namely the unsafe major error-free rate. Contemporary medical LLM benchmark literature emphasizes that not all errors are clinically equivalent, particularly in open-ended clinical tasks [9,10,12]. In the safety-and-effectiveness benchmark study by Wang et al [10], performance was reported to decline by an average of 13.3% in high-risk clinical scenarios. Our findings confirm this general observation in the context of cervical screening; more importantly, however, they demonstrate concretely at which decision nodes and through which error patterns this decline emerges. The study thus supports the view that, in medical LLM evaluation, the capacity to avoid generating harmful mismanagement recommendations is a clinically more meaningful metric than average accuracy alone.

The most striking finding of this study was that GPT-5.3 produced no unsafe major errors under the guideline-directed prompt package and achieved an exact concordance rate of 98.3% within this benchmark. This rate refers to performance on a purposively constructed scenario set that deliberately oversamples complex and history-dependent decision nodes, and it is not a population-level estimate of clinical safety. Gemini 3 Flash reached a safety rate of 98.9% with the guideline-directed prompt package, thereby approaching GPT-5.3, but declined to 80% under baseline conditions. This finding indicates that Gemini 3 Flash reached a high unsafe major error-free rate within this benchmark when appropriately guided, but that this capacity is strongly dependent on prompt configuration. DeepSeek V3.2 exhibited the lowest performance in both arms and failed to exceed a safety rate of 75% even under the guideline-directed prompt package. This result demonstrates that while the guideline-directed prompt package improves performance, it is insufficient on its own when model-specific performance limitations are substantial.

The effect of prompt strategy is among the more directly actionable findings of this study. In the GEE analysis, the guideline-directed prompt package increased the likelihood of an unsafe major error-free response 3.8-fold (OR 3.76, 95% CI 2.45-5.77) and the likelihood of exact concordance 8.3-fold (OR 8.27, 95% CI 4.14-16.52). The guideline-directed prompt package was therefore associated with substantially improved benchmark performance, after adjustment for model and scenario complexity. Because the package varied 3 components jointly—naming the guideline, directing risk-based reasoning, and requesting a structured output—the design cannot attribute this association to output structure alone. Kuerbanjiang et al [16] likewise observed that prompting could improve performance in cervical cancer management; however, that effect was reported at a descriptive level rather than through a controlled comparison. Our study quantified the prompt-package effect in a cluster-aware GEE model and in a complementary scenario-level paired analysis. Absolute pp improvements differed across models in that complementary analysis; however, the formal interaction test for exact concordance was not statistically significant on the odds scale, and the corresponding interaction model for the safety outcome was affected by separation. The increase in the unsafe major error-free rate from 80% to 98.9% in Gemini 3 Flash is consistent with the prompt package constraining the response toward the guideline framework, although the mechanism was not directly measured. These findings support further evaluation of standardized, guideline-directed prompt templates in clinical decision support applications of LLMs.

Scenario complexity showed the largest association with the unsafe major error rate in this study. The overall unsafe major error rate was only 3.1% in low-complexity scenarios but rose to 29% in high-complexity scenarios. In the cluster-aware analysis, high complexity was associated with a marked reduction in the likelihood of an unsafe major error-free response compared with low complexity (OR 0.05; 95% CI 0.01-0.24), and with a still larger reduction in the likelihood of exact concordance (OR 0.005; 95% CI 0.001-0.032). The difference between medium and low complexity did not reach significance for the safety outcome after adjustment for within-scenario clustering (OR 0.30; 95% CI 0.07-1.37), so the gradient is driven principally by the high-complexity stratum. These findings are consistent with those of Wang et al [10], who demonstrated decreased LLM performance in high-risk scenarios, while revealing a more pronounced complexity-performance gradient specific to cervical screening management. This observation is also congruent with the inherent nature of the ASCCP risk-based framework: because the same current result may require entirely different initial management depending on prior history, correct management depends not on memorizing test-result patterns but on dynamically integrating risk context [1-3]. In other words, the core vulnerability of LLMs in this domain may reflect not merely a lack of knowledge but a deficiency in risk-based contextual reasoning. Clinically, this finding is of critical importance, as undermanagement of a high-risk patient may lead to serious diagnostic delay, whereas overmanagement may result in unnecessary invasive procedures. Accordingly, if LLMs are to be considered for clinical decision support, the definition of complexity-stratified safety thresholds appears warranted.

Error subtype analysis represents one of the most original contributions of this study to the existing literature. Whereas previous studies largely evaluated errors within a simple correct/incorrect framework [15,16,18], the present study classified unsafe major errors into clinically meaningful subtypes and demonstrated that different models exhibit distinct error profiles. The most frequently observed error type was undermanagement (39.4%), suggesting that the overall tendency of LLMs is not toward excessive caution but toward insufficient management. Genotype misinterpretation (29.1%) was identified as the second most common subtype, indicating that the models did not adequately internalize the exception rules specific to HPV 16/18. History neglect (22.4%) accounted for all 8 errors produced by GPT-5.3, revealing that even the best-performing model may at times fail to integrate prior screening history into risk estimation. Notably, overmanagement errors were observed exclusively in DeepSeek V3.2, making it the only model to produce both undermanagement and overmanagement errors. Taken together, these 3 dominant error clusters suggest that the central challenge of LLM performance in cervical screening management lies not merely in recognizing test labels but in integrating past and present information within a coherent risk-based reasoning framework. Risk-based and history-dependent domains such as ASCCP therefore represent particularly informative yet high-risk testing environments for LLMs. These differentiated error profiles provide valuable information for the future development of model-specific improvement strategies.

Our intramodel consistency findings are consistent with recent multirun studies. Stelling et al [13] reported that LLM performance on nuclear medicine board examinations varied across repetitions, and Güler et al [14] described similar inconsistency patterns in hand fracture diagnosis. In our study, GPT-5.3 demonstrated complete consistency in 95% to 98.3% of scenarios, whereas DeepSeek V3.2 remained in the range of 86.7% to 93.3%. These findings support the view that accuracy and consistency should be evaluated as distinct constructs, and that reliability as a clinical decision support tool should depend not solely on single-run accuracy but also on reproducible performance.

The exploratory arm 3 findings suggest that checklist-based self-verification demonstrates limited efficacy in its current form. Correction was observed in 16.7% of DeepSeek V3.2 responses and in 8.3% of Gemini 3 Flash responses; formal significance testing was not performed for this arm because the number of discordant scenarios is too small to support inference. More importantly, no model demonstrated a reverse transition in which a previously safe response deteriorated to the unsafe category after checklist review. This asymmetric finding suggests that self-verification mechanisms did not produce deterioration in this exploratory arm but do not yet function as a dependable safety net in their present form. Nevertheless, because this arm was applied to only 12 challenge scenarios for exploratory purposes, the results should be interpreted with caution.

### Comparison With Prior Work

When compared with the existing LLM literature in the cervical cancer domain, our findings diverge at several important points. Pavone et al [18] tested ChatGPT 4.0, DeepSeek R1, and Gemini 2.0 against ESGO guidelines and reported that all models demonstrated suboptimal accuracy; the effect of prompting was not evaluated in a controlled manner in that study. In our benchmark, Gemini 3 Flash achieved a 98.9% unsafe major error-free rate under the guideline-directed prompt package, although its exact concordance rate in the same condition was 93.9%. Direct numerical comparison with Pavone et al [18] should be interpreted cautiously, because their highest quality score denotes complete correctness and therefore corresponds more closely to our exact concordance tier than to the absence of unsafe major errors. Our study nonetheless presents a more differentiated picture, in that high benchmark performance was attainable for some models under a guideline-directed prompt package. Yurtcu et al [15] reported that ChatGPT showed acceptable performance on general knowledge questions but reduced accuracy on guideline-based questions; our findings support this observation and further quantify the performance decline with increasing complexity. The favorable usability findings of Angyal et al [17] regarding a customized GPT model for patient education provide an additional perspective suggesting that informational and decision-support applications of LLMs carry different safety requirements. The findings of Ong et al [12] that an LLM improved the accuracy of medication chart review when used alongside pharmacists are aligned with the central message of our study: LLMs should be positioned not as autonomous tools but as structured decision support systems under clinician supervision.

A further question is what a generative model adds over a deterministic risk calculator for this specific task. The ASCCP framework is itself a rule-based algorithm, and computable versions of it are already being developed: the Centers for Disease Control and Prevention has pursued a multiyear initiative to translate narrative cervical screening guidelines into computable form for integration into electronic health record systems, explicitly motivated by the observation that the complexity and frequent updating of current evidence-based guidelines make it difficult for clinicians to keep pace [26]. A deterministic calculator is reproducible by construction and cannot drift between runs, and for the final risk computation it is likely to be more dependable than a generative model. The potential contribution of an LLM lies elsewhere: extracting the relevant parameters from unstructured clinical narrative, operating where no structured decision-support infrastructure exists, and explaining the basis of a recommendation in natural language. Our findings indicate that this flexibility carries a safety cost precisely at the history-dependent decision nodes where the extraction task is hardest, which argues for pairing rather than substitution.

### Strengths and Limitations

Several strengths of this study should be noted. First, the evaluation focused on a single risk-based management system—the ASCCP framework—providing a more defensible gold standard compared with broad question sets combining heterogeneous guidelines [1-6]. Second, the study used an open-ended, scenario-based design aimed at overcoming the limitations of multiple-choice examination formats [9,10]. Third, the safety-centered error classification allowed the separation of accuracy from harm potential. Fourth, the use of 3 independent repetitions enabled the assessment of intramodel consistency, an approach methodologically aligned with recent studies demonstrating that accuracy and stability are not necessarily equivalent [13,14]. Finally, 2 specialist evaluators scored the outputs independently, and the high κ values support internal consistency of the scoring process.

Several limitations should also be acknowledged. First, the 2 evaluators were not independent of gold-standard construction: both had participated in developing the scenarios and the answer key, and individuals who author an answer key and then apply it are structurally exposed to confirmation bias, particularly at borderline decisions. The interrater κ demonstrates internal consistency of the rating team rather than independence from the constructed gold standard. Disagreement was asymmetric rather than random, and the consensus classification coincided with the first evaluator’s independent score in 116 of the 120 discordant observations. In a post hoc sensitivity analysis, the primary end point was recomputed separately from each evaluator’s independent scores; rates differed by at most 2.8 pp, and neither the model hierarchy nor the direction of the prompt-package effect changed. As a further check, all 60 prespecified gold-standard decisions were verified post hoc against the cited ASCCP guidance; this is source verification of the answer key and not an independent external validation.

Second, the verbatim model outputs were not prospectively archived as part of the study dataset. Because queries were conducted in independent, non–account-linked web sessions, no recoverable session history was subsequently available. Consequently, the mapping from response text to classification category cannot now be independently readjudicated. The coded observation-level dataset (Multimedia Appendix 10), the gold-standard rubric with its source references (Multimedia Appendix 1), the prompt templates (Multimedia Appendix 3), and the analysis code (Multimedia Appendix 6) are provided, but this does not substitute for the original response texts, and this is the principal transparency limitation of the study.

Third, no query-level version identifier, API request identifier, or pinned model checkpoint was recorded, so the exact server-side state of the tested systems cannot be verified or reproduced by another team. This is distinct from, and more consequential than, the general observation that commercial models evolve over time: the latter limits generalization to later versions, whereas the former limits verification of what was actually tested. All models were queried in their free, default, nonreasoning consumer configuration, and the reported rates should not be generalized to reasoning-enabled, retrieval-augmented, or enterprise-configured deployments of the same model families, which may perform differently.

Fourth, scenario complexity and error subtype were each assigned once by investigator consensus, without an independent reliability assessment. No formal reliability coefficient can be computed for the complexity classification because independent ratings were not recorded. Minor and major deviation definitions were applied by consensus during evaluation rather than formalized in a separate written adjudication document. Fifth, synthetic scenarios may not capture all nuances of real-world clinical practice; however, this approach enabled controlled comparison under standardized conditions. Sixth, the scenario set deliberately oversamples complex and history-dependent decision nodes and is not a population-representative case mix, so the reported rates should be read as benchmark performance rather than as population-level safety estimates. Seventh, the study focused exclusively on initial management decisions and did not encompass other components of the ASCCP ecosystem, such as postcolposcopy surveillance and posttreatment follow-up. Eighth, ASCCP is a United States-specific guideline and the international generalizability of the findings is limited; however, the core principles of risk-based management are increasingly being adopted globally. Ninth, the study compared only LLMs and did not include a direct human clinician comparator arm for the same scenarios; although clinicians have previously been shown to experience difficulties with risk-based cervical screening management [7], the absence of a direct human-model comparison represents a deliberate limitation of this study’s scope. Finally, while the 60 scenarios systematically covered the key decision nodes of the ASCCP framework, they do not represent all possible clinical combinations.

Despite these limitations, the study delivers important practical messages regarding LLM performance in cervical screening management. Even in the best-case scenario, safety levels are sensitive to both model selection and prompt design; in the worst-case scenario, history-dependent and high-complexity decision nodes can generate clinically meaningful risk. Future research should therefore focus on 3 directions: first, broader ASCCP coverage encompassing postcolposcopy and posttreatment follow-up domains; second, human-AI interactive workflow studies involving real clinical users; and third, controlled evaluation of self-verification, checklist-based, and retrieval-augmented approaches. Such studies will help clarify the extent to which LLMs can be transformed into safe and useful assistive tools in cervical screening management.

### Conclusions

This scenario-based benchmark study demonstrated that the guideline concordance and safety-oriented benchmark performance of LLMs in the initial management of abnormal cervical screening results according to the 2019 ASCCP risk-based approach vary substantially by model, prompt strategy, and scenario complexity. GPT-5.3 demonstrated the highest benchmark safety and exact concordance performance, particularly under the guideline-directed prompt package; Gemini 3 Flash showed meaningful improvement but retained a prompt-dependent performance profile; and DeepSeek V3.2 showed the lowest benchmark performance under both prompt conditions.

The guideline-directed prompt package was associated with improved performance on both the unsafe major error-free rate and exact guideline concordance, after adjustment for model and scenario complexity. In contrast, performance deteriorated markedly with increasing scenario complexity, with the error burden rising particularly in history-dependent, genotype-sensitive, and multistep decision scenarios. The predominance of undermanagement, genotype misinterpretation, and history neglect as the most frequent error types suggests that the principal vulnerability of LLMs in this domain lies not only in incomplete knowledge but also in insufficient risk-based contextual reasoning.

These findings indicate that LLMs may have value as structured, clinician-supervised decision support tools in cervical screening management, but that they have not yet reached a sufficient level of safety for autonomous clinical use. Future studies incorporating broader ASCCP coverage, human-AI interactive workflows, and structured verification strategies will be critical for evaluating the safe clinical integration of these tools.

## Appendix

Multimedia Appendix 1 Prespecified gold-standard management decision for each of the 60 clinical scenarios, with its American Society for Colposcopy and Cervical Pathology (ASCCP) source reference and post hoc source verification.

Multimedia Appendix 2 Full list of the 60 clinical scenarios used for benchmark evaluation, with category and complexity classification.

Multimedia Appendix 3 Verbatim prompt templates used in the 3 study arms.

Multimedia Appendix 4 Illustrative scoring example demonstrating the 3-tier classification system.

Multimedia Appendix 5 Supplementary tables: interrater reliability, exploratory arm 3, unadjusted response-level CIs, pairwise model comparisons, and sensitivity analyses.

Multimedia Appendix 6 Analysis code used to generate the reported statistics, with a README describing which script produces each table.

Multimedia Appendix 7 CHART checklist.

Multimedia Appendix 8 Response classification heatmap by scenario, model, and prompt arm, annotated by complexity tier and coverage group.

Multimedia Appendix 9 Intramodel response consistency across 3 repetitions.

Multimedia Appendix 10 Coded observation-level evaluation dataset for the primary analysis and the exploratory arm 3.

## Abbreviations

AGC: atypical glandular cells
ASCCP: American Society for Colposcopy and Cervical Pathology
ASC-H: atypical squamous cells, cannot exclude high-grade squamous intraepithelial lesion
ASC-US: atypical squamous cells of undetermined significance
CHART: Chatbot Assessment Reporting Tool
CIN 3+: cervical intraepithelial neoplasia grade 3 or worse
ESGO: European Society of Gynaecological Oncology
ESP: European Society of Pathology
ESTRO: European Society for Radiotherapy and Oncology
GEE: generalized estimating equation
HPV: human papillomavirus
HSIL: high-grade squamous intraepithelial lesion
ICC: intraclass correlation coefficient
LLM: large language model
LSIL: low-grade squamous intraepithelial lesion
NILM: negative for intraepithelial lesion or malignancy
OR: odds ratio
pp: percentage points
TRIPOD-LLM: Transparent Reporting of a Multivariable Prediction Model for Individual Prognosis or Diagnosis–Large Language Models

## Data Availability

The coded observation-level evaluation dataset and the exploratory arm 3 dataset, the gold-standard rubric with its ASCCP source references, the verbatim prompt templates, and the analysis code are provided as Multimedia Appendices 10, 1, 3, and 6, respectively. The verbatim model response texts are not available. All queries were conducted through publicly available web interfaces in independent, non–account-linked sessions; the response texts were not prospectively archived as part of the study dataset and no recoverable session history was subsequently available. These materials therefore permit reproduction of every reported count, proportion, and test statistic, and inspection of the answer key, but not independent readjudication of the mapping from response text to classification category.
