# Supplementary material for: Safety-Oriented Benchmarking of Large Language Models in Risk-Based Management of Abnormal Cervical Screening Results: Scenario-Based Benchmark Study
Source: J Med Internet Res. 2026 Sep 22;28:e98131. doi: 10.2196/98131 (PMC13643256; doi:10.2196/98131)
Supplement: Supplementary file 1 [file jmir_v28i1e98131_app2.docx]

**Multimedia Appendix 2. Full List of Clinical Scenarios Used for Benchmark Evaluation**

| **No** | **Category** | **Complexity** | **Clinical Vignette** |
| --- | --- | --- | --- |
| 1 | Core | Low | A 32-year-old woman presents for cervical cancer screening. No previous screening history is available. The current cotest result shows HPV negative and cytology reported as NILM (negative for intraepithelial lesion or malignancy). She has no history of cervical biopsy or treatment. What is the recommended initial management according to the 2019 ASCCP guidelines? |
| 2 | Core | Low | A 45-year-old woman presents for cervical cancer screening. No previous screening history is available. The current cotest result shows HPV negative and cytology reported as ASC-US (atypical squamous cells of undetermined significance). She has no history of cervical biopsy or treatment. What is the recommended initial management according to the 2019 ASCCP guidelines? |
| 3 | Core | Medium | A 38-year-old woman presents for cervical cancer screening. No previous screening history is available. The current cotest result shows HPV negative and cytology reported as LSIL (low-grade squamous intraepithelial lesion). She has no history of cervical biopsy or treatment. What is the recommended initial management according to the 2019 ASCCP guidelines? |
| 4 | Core | Low | A 30-year-old woman presents for cervical cancer screening. No previous screening history is available. The current cotest result shows HPV positive for a high-risk type other than HPV 16 or HPV 18, and cytology reported as NILM. She has no history of cervical biopsy or treatment. What is the recommended initial management according to the 2019 ASCCP guidelines? |
| 5 | Core | Low | A 42-year-old woman presents for cervical cancer screening. No previous screening history is available. The current cotest result shows HPV positive for a high-risk type other than HPV 16 or HPV 18, and cytology reported as ASC-US. She has no history of cervical biopsy or treatment. What is the recommended initial management according to the 2019 ASCCP guidelines? |
| 6 | Core | Low | A 36-year-old woman presents for cervical cancer screening. No previous screening history is available. The current cotest result shows HPV positive for a high-risk type other than HPV 16 or HPV 18, and cytology reported as LSIL. She has no history of cervical biopsy or treatment. What is the recommended initial management according to the 2019 ASCCP guidelines? |
| 7 | Core | Low | A 34-year-old woman presents for cervical cancer screening. No previous screening history is available. The current cotest result shows HPV 16 positive and cytology reported as NILM. She has no history of cervical biopsy or treatment. What is the recommended initial management according to the 2019 ASCCP guidelines? |
| 8 | Core | Low | A 40-year-old woman presents for cervical cancer screening. No previous screening history is available. The current cotest result shows HPV 16 positive and cytology reported as ASC-US. She has no history of cervical biopsy or treatment. What is the recommended initial management according to the 2019 ASCCP guidelines? |
| 9 | Core | High | A 33-year-old woman presents for cervical cancer screening. No previous screening history is available. The current cotest result shows HPV 18 positive and cytology reported as NILM. She has no history of cervical biopsy or treatment. What is the recommended initial management according to the 2019 ASCCP guidelines? |
| 10 | Core | Medium | A 40-year-old woman presents for cervical cancer screening. No previous screening history is available. The current cotest result shows HPV 18 positive and cytology reported as ASC-US. She has no history of cervical biopsy or treatment. What is the recommended initial management according to the 2019 ASCCP guidelines? |
| 11 | Core | Medium | A 28-year-old woman presents for cervical cancer screening. No previous screening history is available. The current cotest result shows HPV positive for a high-risk type other than HPV 16 or HPV 18, and cytology reported as ASC-H (atypical squamous cells, cannot exclude HSIL). She has no history of cervical biopsy or treatment. What is the recommended initial management according to the 2019 ASCCP guidelines? |
| 12 | Core | High | A 55-year-old woman presents for cervical cancer screening. No previous screening history is available. The current cotest result shows HPV negative and cytology reported as ASC-H. She has no history of cervical biopsy or treatment. What is the recommended initial management according to the 2019 ASCCP guidelines? |
| 13 | Core | Medium | A 34-year-old woman presents for cervical cancer screening. No previous screening history is available. The current cotest result shows HPV positive for a high-risk type other than HPV 16 or HPV 18, and cytology reported as HSIL (high-grade squamous intraepithelial lesion). She has no concerns about the potential effect of treatment on future pregnancy outcomes. She has no history of cervical biopsy or treatment. What is the recommended initial management according to the 2019 ASCCP guidelines? |
| 14 | Core | High | A 40-year-old woman presents for cervical cancer screening. No previous screening history is available. The current cotest result shows HPV negative and cytology reported as HSIL. She has no concerns about the potential effect of treatment on future pregnancy outcomes. She has no history of cervical biopsy or treatment. What is the recommended initial management according to the 2019 ASCCP guidelines? |
| 15 | Core | High | A 32-year-old woman presents for cervical cancer screening. No previous screening history is available. The current cotest result shows HPV 16 positive and cytology reported as HSIL. She has no concerns about the potential effect of treatment on future pregnancy outcomes. She has no history of cervical biopsy or treatment. What is the recommended initial management according to the 2019 ASCCP guidelines? |
| 16 | Core | Medium | A 38-year-old woman presents for cervical cancer screening. No previous screening history is available. The current cotest result shows HPV 18 positive and cytology reported as HSIL. She has no concerns about the potential effect of treatment on future pregnancy outcomes. She has no history of cervical biopsy or treatment. What is the recommended initial management according to the 2019 ASCCP guidelines? |
| 17 | Core | Medium | A 52-year-old woman presents for cervical cancer screening. No previous screening history is available. The current cotest result shows HPV positive for a high-risk type other than HPV 16 or HPV 18, and cytology reported as atypical glandular cells, not otherwise specified (AGC-NOS). She has no history of cervical biopsy or treatment. What is the recommended initial management according to the 2019 ASCCP guidelines? |
| 18 | Core | Medium | A 29-year-old woman presents for cervical cancer screening. No previous screening history is available. The current cotest result shows HPV positive for a high-risk type other than HPV 16 or HPV 18, and cytology reported as AGC-NOS. She has no abnormal uterine bleeding, no obesity, and no conditions suggesting chronic anovulation. She has no history of cervical biopsy or treatment. What is the recommended initial management according to the 2019 ASCCP guidelines? |
| 19 | Core | High | A 50-year-old woman presents for cervical cancer screening. No previous screening history is available. The current cotest result shows HPV negative and cytology reported as AGC-NOS. She has no history of cervical biopsy or treatment. What is the recommended initial management according to the 2019 ASCCP guidelines? |
| 20 | Core | High | A 44-year-old woman presents for cervical cancer screening. No previous screening history is available. The current cotest result shows HPV positive for a high-risk type other than HPV 16 or HPV 18, and cytology reported as atypical glandular cells, favor neoplasia. She has no history of cervical biopsy or treatment. What is the recommended initial management according to the 2019 ASCCP guidelines? |
| 21 | Core | Low | A 50-year-old woman presents for cervical cancer screening. Her previous cotest 3 years ago showed HPV negative and cytology NILM. The current cotest result shows HPV negative and cytology NILM. She has no history of cervical biopsy or treatment. What is the recommended initial management according to the 2019 ASCCP guidelines? |
| 22 | Core | Low | A 35-year-old woman presents for cervical cancer screening. Her previous cotest 4 years ago showed HPV negative and cytology NILM. The current cotest result shows HPV negative and cytology ASC-US. She has no history of cervical biopsy or treatment. What is the recommended initial management according to the 2019 ASCCP guidelines? |
| 23 | Core | Medium | A 38-year-old woman presents for cervical cancer screening. Her previous cotest 3 years ago showed HPV negative and cytology NILM. The current cotest result shows HPV positive for a high-risk type other than HPV 16 or HPV 18, and cytology NILM. She has no history of cervical biopsy or treatment. What is the recommended initial management according to the 2019 ASCCP guidelines? |
| 24 | Core | High | A 35-year-old woman presents for cervical cancer screening. Her previous cotest 4 years ago showed HPV negative and cytology NILM. The current cotest result shows HPV positive for a high-risk type other than HPV 16 or HPV 18, and cytology ASC-US. She has no history of cervical biopsy or treatment. What is the recommended initial management according to the 2019 ASCCP guidelines? |
| 25 | Core | High | A 40-year-old woman presents for cervical cancer screening. Her previous cotest 4 years ago showed HPV negative and cytology NILM. The current cotest result shows HPV positive for a high-risk type other than HPV 16 or HPV 18, and cytology LSIL. She has no history of cervical biopsy or treatment. What is the recommended initial management according to the 2019 ASCCP guidelines? |
| 26 | Core | High | A 42-year-old woman presents for cervical cancer screening. Her previous cotest 3 years ago showed HPV negative and cytology NILM. The current cotest result shows HPV 16 positive and cytology NILM. She has no history of cervical biopsy or treatment. What is the recommended initial management according to the 2019 ASCCP guidelines? |
| 27 | Core | High | A 36-year-old woman presents for cervical cancer screening. Her previous cotest 3 years ago showed HPV negative and cytology NILM. The current cotest result shows HPV 18 positive and cytology NILM. She has no history of cervical biopsy or treatment. What is the recommended initial management according to the 2019 ASCCP guidelines? |
| 28 | Core | Medium | A 38-year-old woman presents for cervical cancer screening. Her previous cotest 4 years ago showed HPV negative and cytology NILM. The current cotest result shows HPV positive for a high-risk type other than HPV 16 or HPV 18, and cytology ASC-H. She has no history of cervical biopsy or treatment. What is the recommended initial management according to the 2019 ASCCP guidelines? |
| 29 | Core | Low | A 30-year-old woman undergoes primary HPV screening. The result shows HPV negative. No cytology was performed. She has no previous screening history available and no history of cervical biopsy or treatment. What is the recommended initial management according to the 2019 ASCCP guidelines? |
| 30 | Core | Low | A 35-year-old woman undergoes primary HPV screening. The result shows HPV positive for a high-risk type other than HPV 16 or HPV 18. Reflex cytology from the same specimen shows NILM. She has no previous screening history available and no history of cervical biopsy or treatment. What is the recommended initial management according to the 2019 ASCCP guidelines? |
| 31 | Core | Low | A 45-year-old woman undergoes primary HPV screening. The result shows HPV positive for a high-risk type other than HPV 16 or HPV 18. Reflex cytology from the same specimen shows ASC-US. She has no previous screening history available and no history of cervical biopsy or treatment. What is the recommended initial management according to the 2019 ASCCP guidelines? |
| 32 | Core | Medium | A 30-year-old woman undergoes primary HPV screening. The result shows HPV 16 positive. Reflex cytology from the same specimen shows NILM. She has no previous screening history available and no history of cervical biopsy or treatment. What is the recommended initial management according to the 2019 ASCCP guidelines? |
| 33 | Core | High | A 38-year-old woman undergoes primary HPV screening. The result shows HPV 16 positive. Reflex cytology from the same specimen shows HSIL. She has no previous screening history available, no history of cervical biopsy or treatment, and no concerns about the potential effect of treatment on future pregnancy outcomes. What is the recommended initial management according to the 2019 ASCCP guidelines? |
| 34 | Core | Medium | A 42-year-old woman undergoes primary HPV screening. The result shows HPV 18 positive. Reflex cytology from the same specimen shows NILM. She has no previous screening history available and no history of cervical biopsy or treatment. What is the recommended initial management according to the 2019 ASCCP guidelines? |
| 35 | Core | Medium | A 35-year-old woman presents for cervical cancer screening. The current cotest result shows HPV negative and cytology reported as unsatisfactory. She has no previous screening history available and no history of cervical biopsy or treatment. What is the recommended initial management according to the 2019 ASCCP guidelines? |
| 36 | Core | Medium | A 30-year-old woman presents for cervical cancer screening. The current cotest result shows HPV positive for a high-risk type other than HPV 16 or HPV 18, and cytology reported as unsatisfactory. She has no previous screening history available and no history of cervical biopsy or treatment. What is the recommended initial management according to the 2019 ASCCP guidelines? |
| 37 | Core | Medium | A 32-year-old woman presents for cervical cancer screening. The current cotest result shows HPV 16 positive and cytology reported as unsatisfactory. She has no previous screening history available and no history of cervical biopsy or treatment. What is the recommended initial management according to the 2019 ASCCP guidelines? |
| 38 | Core | Medium | A 35-year-old woman presents for cervical cancer screening with cytology alone. The cytology result is NILM, but the report notes absent endocervical cells and transformation zone component. No HPV test was performed. She has no previous screening history available and no history of cervical biopsy or treatment. What is the recommended initial management according to the 2019 ASCCP guidelines? |
| 39 | Core | Medium | A 48-year-old postmenopausal asymptomatic woman presents for cervical cancer screening. The current cotest result shows HPV negative and cytology NILM with benign endometrial cells reported. She has no history of cervical biopsy or treatment. What is the recommended initial management according to the 2019 ASCCP guidelines? |
| 40 | Core | Low | A 33-year-old premenopausal asymptomatic woman with regular menstrual cycles presents for cervical cancer screening. The current cotest result shows HPV negative and cytology NILM with benign endometrial cells reported. She has no history of cervical biopsy or treatment. What is the recommended initial management according to the 2019 ASCCP guidelines? |
| 41 | Core | Low | A 26-year-old woman presents for cervical cancer screening. No previous screening history is available. The current cotest result shows HPV positive for a high-risk type other than HPV 16 or HPV 18, and cytology ASC-US. She has no history of cervical biopsy or treatment. What is the recommended initial management according to the 2019 ASCCP guidelines? |
| 42 | Core | Low | A 62-year-old woman presents for cervical cancer screening. No previous screening history is available. The current cotest result shows HPV positive for a high-risk type other than HPV 16 or HPV 18, and cytology NILM. She has no history of cervical biopsy or treatment. What is the recommended initial management according to the 2019 ASCCP guidelines? |
| 43 | Core | Medium | A 29-year-old woman presents for cervical cancer screening. No previous screening history is available. The current cotest result shows HPV 16 positive and cytology LSIL. She has no history of cervical biopsy or treatment. What is the recommended initial management according to the 2019 ASCCP guidelines? |
| 44 | Core | Medium | A 50-year-old woman presents for cervical cancer screening. No previous screening history is available. The current cotest result shows HPV positive for a high-risk type other than HPV 16 or HPV 18, and cytology HSIL. She has no concerns about the potential effect of treatment on future pregnancy outcomes. She has no history of cervical biopsy or treatment. What is the recommended initial management according to the 2019 ASCCP guidelines? |
| 45 | Core | Medium | A 64-year-old woman presents for cervical cancer screening. No previous screening history is available. The current cotest result shows HPV 18 positive and cytology NILM. She has no history of cervical biopsy or treatment. What is the recommended initial management according to the 2019 ASCCP guidelines? |
| 46 | Core | Low | A 27-year-old woman presents for cervical cancer screening. No previous screening history is available. The current cotest result shows HPV negative and cytology LSIL. She has no history of cervical biopsy or treatment. What is the recommended initial management according to the 2019 ASCCP guidelines? |
| 47 | Core | Medium | A 45-year-old woman presents for cervical cancer screening. No previous screening history is available. The current cotest result shows HPV positive for a high-risk type other than HPV 16 or HPV 18, and cytology reported as AGC-NOS. She has no history of cervical biopsy or treatment. What is the recommended initial management according to the 2019 ASCCP guidelines? |
| 48 | Core | High | A 31-year-old obese woman with irregular menstrual cycles suggestive of chronic anovulation presents for cervical cancer screening. No previous screening history is available. The current cotest result shows HPV positive for a high-risk type other than HPV 16 or HPV 18, and cytology reported as AGC-NOS. She has no history of cervical biopsy or treatment. What is the recommended initial management according to the 2019 ASCCP guidelines? |
| 49 | Challenge | High | A 37-year-old woman presents for cervical cancer screening. No previous screening history is available. The current cotest result shows HPV positive for a high-risk type other than HPV 16 or HPV 18, and cytology ASC-US. She has no history of cervical biopsy or treatment. What is the recommended initial management according to the 2019 ASCCP guidelines? |
| 50 | Challenge | High | A 39-year-old woman presents for cervical cancer screening. No previous screening history is available. The current cotest result shows HPV positive for a high-risk type other than HPV 16 or HPV 18, and cytology LSIL. She has no history of cervical biopsy or treatment. What is the recommended initial management according to the 2019 ASCCP guidelines? |
| 51 | Challenge | High | A 35-year-old woman presents for cervical cancer screening. Her previous screening 3 years ago was cytology alone, which showed NILM; no HPV test was performed at that time. The current cotest result shows HPV positive for a high-risk type other than HPV 16 or HPV 18, and cytology ASC-US. She has no history of cervical biopsy or treatment. What is the recommended initial management according to the 2019 ASCCP guidelines? |
| 52 | Challenge | High | A 42-year-old woman presents for cervical cancer screening. Her previous cotest 4 years ago showed HPV negative and cytology NILM. The current cotest result shows HPV 16 positive and cytology ASC-US. She has no history of cervical biopsy or treatment. What is the recommended initial management according to the 2019 ASCCP guidelines? |
| 53 | Challenge | High | A 38-year-old woman presents for cervical cancer screening. Her previous cotest 3 years ago showed HPV negative and cytology NILM. The current cotest result shows HPV 18 positive and cytology NILM. She has no history of cervical biopsy or treatment. What is the recommended initial management according to the 2019 ASCCP guidelines? |
| 54 | Challenge | High | A 40-year-old woman who has never undergone cervical cancer screening presents for the first time. The current cotest result shows HPV positive (genotype not specified) and cytology HSIL. She has no concerns about the potential effect of treatment on future pregnancy outcomes. What is the recommended initial management according to the 2019 ASCCP guidelines? |
| 55 | Challenge | High | A 30-year-old woman presents for cervical cancer screening. No previous screening history is available. The current cotest result shows HPV 16 positive and cytology HSIL. She has significant concerns about the potential effect of treatment on future pregnancy outcomes. She has no history of cervical biopsy or treatment. What is the recommended initial management according to the 2019 ASCCP guidelines? |
| 56 | Challenge | High | A 28-year-old woman presents for cervical cancer screening. No previous screening history is available. The current cotest result shows HPV positive for a high-risk type other than HPV 16 or HPV 18, and cytology HSIL. She has concerns about the potential effect of treatment on future pregnancy outcomes. She has no history of cervical biopsy or treatment. What is the recommended initial management according to the 2019 ASCCP guidelines? |
| 57 | Challenge | High | A 45-year-old woman presents for cervical cancer screening. No previous screening history is available. The current cotest result shows HPV negative and cytology reported as atypical glandular cells, favor neoplasia. She has no history of cervical biopsy or treatment. What is the recommended initial management according to the 2019 ASCCP guidelines? |
| 58 | Challenge | High | A 52-year-old woman presents for cervical cancer screening. No previous screening history is available. The current cotest result shows HPV positive for a high-risk type other than HPV 16 or HPV 18, and cytology reported as atypical endometrial cells. She has no history of cervical biopsy or treatment. What is the recommended initial management according to the 2019 ASCCP guidelines? |
| 59 | Challenge | High | A 33-year-old woman undergoes primary HPV screening. The result shows HPV 16 positive. However, reflex cytology from the same laboratory specimen was not feasible. She has no previous screening history available and no history of cervical biopsy or treatment. What is the recommended initial management according to the 2019 ASCCP guidelines? |
| 60 | Challenge | Medium | A 40-year-old woman presents for cervical cancer screening. Her previous cytology 6 months ago was reported as unsatisfactory. The current repeat cytology is again reported as unsatisfactory. HPV testing was not performed on either occasion. She has no history of cervical biopsy or treatment. What is the recommended initial management according to the 2019 ASCCP guidelines? |

*Core: standard clinical decision scenarios (n = 48); Challenge: scenarios specifically designed to test history-dependent interpretation, genotype-specific exception rules, borderline risk thresholds, and age-dependent decision nodes (n = 12). Low complexity: management largely determined by current test result alone; Medium complexity: integration of at least one additional clinical variable required; High complexity: guideline exceptions, history-dependent management, or multi-step decision processes involved.*
