# Supplementary material for: Safety-Oriented Benchmarking of Large Language Models in Risk-Based Management of Abnormal Cervical Screening Results: Scenario-Based Benchmark Study
Source: J Med Internet Res. 2026 Sep 22;28:e98131. doi: 10.2196/98131 (PMC13643256; doi:10.2196/98131)
Supplement: Supplementary file 2 [file jmir_v28i1e98131_app3.docx]

**Multimedia Appendix 3. Prompt Templates Used in the Three Study Arms**

This appendix presents the exact prompt templates used for each of the three prompt arms in this study. Each scenario was embedded into the template structure shown below. All prompts were delivered in English via the public web interfaces of the respective models. Each query was initiated in a new, independent chat session with no prior conversational context.

**A1. Arm 1 — Baseline Clinical Prompt**

In this arm, the clinical scenario was presented without any explicit guideline reference or structured clinical directive. The model was asked to provide its management recommendation based on its clinical knowledge. The output format requested a management decision and a brief clinical rationale.

*Example prompt (Scenario 1, Low complexity):*

You are a gynecology specialist. A patient presents to your clinic with the following cervical cancer screening results. Based on your clinical knowledge, provide the recommended initial management for this patient.

A 32-year-old woman presents for cervical cancer screening. No previous screening history is available. The current cotest result shows HPV negative and cytology reported as NILM (negative for intraepithelial lesion or malignancy). She has no history of cervical biopsy or treatment. What is the recommended initial management for this patient?

Please provide:

1. Your recommended management decision (e.g., return to routine screening, repeat testing at a specific interval, colposcopy, or treatment).

2. A brief clinical rationale for your recommendation.

Important: Provide your entire response in plain text only. Do not use tables, bullet points, markdown formatting, bold text, or any special formatting. Write your answer as continuous paragraphs.

At the end of your response, clearly state your final recommendation in the following format:

Final Recommendation: [your management decision]

**A2. Arm 2 — Structured Guideline-Directed Prompt**

In this arm, the same clinical scenario was presented with an explicit reference to the 2019 ASCCP Risk-Based Management Consensus Guidelines and a directive to use the risk-based approach with Clinical Action Thresholds. The output format additionally requested the estimated risk level and the relevant ASCCP guideline section.

*Example prompt (Scenario 1, Low complexity):*

You are a gynecology specialist. A patient presents to your clinic with the following cervical cancer screening results. Using the 2019 ASCCP Risk-Based Management Consensus Guidelines as your reference, provide the recommended initial management for this patient.

Important: Your answer must be based on the risk-based approach defined by the 2019 ASCCP guidelines, which uses a combination of current test results and prior screening history to estimate the immediate and 5-year risk of CIN 3+ and assigns management according to defined Clinical Action Thresholds.

A 32-year-old woman presents for cervical cancer screening. No previous screening history is available. The current cotest result shows HPV negative and cytology reported as NILM (negative for intraepithelial lesion or malignancy). She has no history of cervical biopsy or treatment. What is the recommended initial management according to the 2019 ASCCP guidelines?

Please provide:

1. Your recommended management decision according to the 2019 ASCCP guidelines (e.g., return to routine screening in 5 years, repeat HPV-based testing in 1 year, repeat HPV-based testing in 3 years, colposcopy, expedited treatment, or other specific recommendation).

2. The estimated risk level that supports this recommendation (if applicable).

3. The relevant ASCCP guideline section or Clinical Action Threshold that applies.

Important: Provide your entire response in plain text only. Do not use tables, bullet points, markdown formatting, bold text, or any special formatting. Write your answer as continuous paragraphs.

At the end of your response, clearly state your final recommendation in the following format:

Final Recommendation: [your management decision]

**A3. Arm 3 — Checklist-Based Self-Verification Prompt (Exploratory)**

This exploratory arm was applied only to the 12 challenge scenarios. In Step 1, the Arm 2 (Structured Guideline-Directed) prompt was sent and the model's initial response was obtained. In Step 2, without opening a new session, the following checklist-based follow-up prompt was sent within the same conversation. The checklist explicitly provided ASCCP Clinical Action Threshold values and genotype-specific exception rules. The model was asked to self-correct if errors were identified. This arm was not included in the primary analysis and was reported as exploratory.

*Step 2 follow-up prompt (sent after receiving the Arm 2 response in the same session):*

Please review your answer above and verify it against the following checklist:

1. Did you consider the patient's prior screening history (or lack thereof) when estimating risk? A prior negative HPV-based test within 5 years reduces CIN 3+ risk by approximately 50%. A prior negative cytology alone does NOT reduce risk.

2. Did you check whether HPV genotype 16 or 18 triggers any exceptions to the standard Clinical Action Thresholds? (HPV 16-positive NILM and HPV 18-positive NILM both warrant colposcopy despite CIN 3+ risk below 4%.)

3. Did you check whether ASC-H cytology warrants colposcopy regardless of HPV result?

4. For HSIL cytology, did you evaluate whether expedited treatment is preferred (CIN 3+ risk >=60%), acceptable (25-59%), or not indicated?

5. For AGC cytology, did you include endocervical sampling and endometrial sampling (if the patient is 35 years or older or has risk factors for endometrial neoplasia)?

6. Is your recommended management consistent with the Clinical Action Thresholds: <0.15% (5-year return), 0.15-0.54% (3-year return), >=0.55% (1-year return), >=4% immediate (colposcopy), 25-59% immediate (expedited treatment acceptable), >=60% immediate (expedited treatment preferred)?

If you find any errors or omissions in your initial answer, please provide a corrected recommendation. If your answer is correct, confirm it.

Important: Provide your entire response in plain text only. Do not use tables, bullet points, markdown formatting, bold text, or any special formatting. Write your answer as continuous paragraphs.

State your final recommendation in the following format:

Final Recommendation: [your management decision]
