# Supplementary material for: Safety-Oriented Benchmarking of Large Language Models in Risk-Based Management of Abnormal Cervical Screening Results: Scenario-Based Benchmark Study
Source: J Med Internet Res. 2026 Sep 22;28:e98131. doi: 10.2196/98131 (PMC13643256; doi:10.2196/98131)
Supplement: Supplementary file 4 [file jmir_v28i1e98131_app5.docx]

**Supplementary Tables**

**Table S1. Inter-rater reliability**

| **Metric** | **Value** |
| --- | --- |
| Total rated responses | 1080 |
| Raw agreement | 960/1080 (88.9%) |
| Cohen kappa, unweighted | 0.770 (95% CI, 0.732–0.807); ASE 0.019 |
| Cohen kappa, linear weighted | 0.839 (95% CI, 0.811–0.867); ASE 0.014 |
| Interpretation, unweighted | Substantial |
| Interpretation, weighted | Almost perfect |

**Cross-classification of the two evaluators**

| **Evaluator 1** | **Evaluator 2: exact correct** | **Evaluator 2: safe but imperfect** | **Evaluator 2: unsafe major error** | **Total** |
| --- | --- | --- | --- | --- |
| Exact correct | 695 | 81 | 0 | 776 |
| Safe but imperfect | 11 | 115 | 13 | 139 |
| Unsafe major error | 0 | 15 | 150 | 165 |
| **Total** | **706** | **211** | **163** | **1080** |

*Confidence intervals were calculated using the asymptotic standard error (ASE) for weighted kappa. A scenario-cluster bootstrap yielded a comparable interval for the linearly weighted coefficient (0.801–0.866). Interpretation follows Landis and Koch, who describe 0.61–0.80 as substantial and 0.81–1.00 as almost perfect while noting that these divisions are arbitrary. Disagreement was asymmetric: 81 responses were classified as exact correct by evaluator 1 and safe but imperfect by evaluator 2, whereas only 11 were classified in the reverse direction.*

**Table S2. Exploratory Arm 3: checklist-based self-verification on the 12 challenge scenarios**

| **Model** | **n** | **Initial exact correct, n (%)** | **Post-checklist exact correct, n (%)** | **Initial unsafe major error, n (%)** | **Post-checklist unsafe major error, n (%)** | **Self-corrected, n (%)** |
| --- | --- | --- | --- | --- | --- | --- |
| GPT-5.3 | 36 | 36 (100.0) | 36 (100.0) | 0 (0.0) | 0 (0.0) | 0 (0.0) |
| Gemini 3 Flash | 36 | 30 (83.3) | 33 (91.7) | 3 (8.3) | 1 (2.8) | 3 (8.3) |
| DeepSeek V3.2 | 36 | 12 (33.3) | 18 (50.0) | 15 (41.7) | 11 (30.6) | 6 (16.7) |

*Arm 3 is exploratory and was not included in the primary analysis. The Arm 2 prompts were re-run in separate independent sessions on the 12 challenge scenarios, so these 108 responses are additional to and not a subset of the 1080 primary-analysis responses; the initial classifications therefore differ from the Arm 2 classifications reported in Table 2. No reverse transitions from safe to unsafe were observed in any model. Formal significance testing is not reported because the number of discordant scenarios is too small to support inference.*

**Table S3. Unadjusted response-level Wilson confidence intervals for the primary outcome**

| **Model** | **Prompt arm** | **Unsafe major error-free rate, %** | **Unadjusted Wilson 95% CI** | **Cluster-adjusted 95% CI (Table 2)** |
| --- | --- | --- | --- | --- |
| GPT-5.3 | Arm 1 | 95.6 | 91.5–97.7 | 87.6–98.5 |
| GPT-5.3 | Arm 2 | 100.0 | 97.9–100.0 | 94.0–100.0 |
| Gemini 3 Flash | Arm 1 | 80.0 | 73.6–85.2 | 69.0–87.8 |
| Gemini 3 Flash | Arm 2 | 98.9 | 96.0–99.7 | 94.0–99.8 |
| DeepSeek V3.2 | Arm 1 | 58.9 | 51.6–65.8 | 46.9–69.9 |
| DeepSeek V3.2 | Arm 2 | 75.0 | 68.2–80.8 | 63.2–84.0 |

*Unadjusted intervals treat each of the 180 responses per cell as an independent observation and are shown for comparison only. Because each scenario was queried three times, the effective sample size is smaller than the nominal sample size and the unadjusted intervals understate sampling uncertainty. The cluster-adjusted intervals reported in Table 2 are the primary estimates.*

**Table S4. Pairwise model comparisons within each prompt arm**

| **Outcome** | **Prompt arm** | **Comparison** | **Odds ratio** | **95% CI** | **P value** |
| --- | --- | --- | --- | --- | --- |
| Unsafe major error-free | Arm 1 | Gemini 3 Flash vs GPT-5.3 | 0.186 | 0.067–0.521 | .001 |
|  | Arm 1 | DeepSeek V3.2 vs GPT-5.3 | 0.067 | 0.021–0.207 | <.001 |
|  | Arm 1 | DeepSeek V3.2 vs Gemini 3 Flash | 0.358 | 0.216–0.594 | <.001 |
|  | Arm 2 | Gemini 3 Flash vs GPT-5.3 | Not estimable | — | — |
|  | Arm 2 | DeepSeek V3.2 vs GPT-5.3 | Not estimable | — | — |
|  | Arm 2 | DeepSeek V3.2 vs Gemini 3 Flash | 0.034 | 0.005–0.229 | .001 |
| Exact concordance | Arm 1 | Gemini 3 Flash vs GPT-5.3 | 0.211 | 0.107–0.416 | <.001 |
|  | Arm 1 | DeepSeek V3.2 vs GPT-5.3 | 0.093 | 0.044–0.201 | <.001 |
|  | Arm 1 | DeepSeek V3.2 vs Gemini 3 Flash | 0.444 | 0.294–0.670 | <.001 |
|  | Arm 2 | Gemini 3 Flash vs GPT-5.3 | 0.260 | 0.073–0.927 | .038 |
|  | Arm 2 | DeepSeek V3.2 vs GPT-5.3 | 0.020 | 0.006–0.071 | <.001 |
|  | Arm 2 | DeepSeek V3.2 vs Gemini 3 Flash | 0.078 | 0.028–0.214 | <.001 |

*Each comparison is a separate generalized estimating equation model restricted to the two models being compared, with scenario as the clustering unit and robust standard errors. Odds ratios below 1 indicate lower odds for the first-named model. With three comparisons per outcome and arm, the Bonferroni-corrected significance threshold is .0167; the Gemini 3 Flash versus GPT-5.3 comparison for exact concordance in Arm 2 (P = .038) does not meet this threshold. The two Arm 2 comparisons involving GPT-5.3 for the unsafe major error-free outcome are not estimable because GPT-5.3 produced no unsafe major errors in that arm; the observed rates were 100.0%, 98.9% and 75.0% for GPT-5.3, Gemini 3 Flash and DeepSeek V3.2 respectively.*

**Table S5. Sensitivity and complementary analyses**

**Model-by-arm interaction, generalized estimating equations**

| **Outcome** | **Interaction term** | **Odds ratio** | **95% CI** | **P value** |
| --- | --- | --- | --- | --- |
| Exact concordance | DeepSeek V3.2 × Arm 2 | 0.325 | 0.075–1.418 | .135 |
| Exact concordance | Gemini 3 Flash × Arm 2 | 2.516 | 0.469–13.483 | .281 |
| Unsafe major error-free | Both terms | Not estimable | — | — |

**Firth penalized logistic regression, sensitivity analysis for separation**

| **Outcome** | **Term** | **Odds ratio** | **Profile penalized likelihood 95% CI** |
| --- | --- | --- | --- |
| Unsafe major error-free | Arm 2 | 18.54 | 2.26–2406.5 |
|  | Gemini 3 Flash × Arm 2 | 1.19 | 0.008–17.16 |
|  | DeepSeek V3.2 × Arm 2 | 0.141 | 0.001–1.255 |
| Exact concordance | Arm 2 | 9.43 | 3.29–36.44 |
|  | Gemini 3 Flash × Arm 2 | 2.59 | 0.567–9.77 |
|  | DeepSeek V3.2 × Arm 2 | 0.339 | 0.080–1.130 |

**Scenario-level paired analysis of the prompt-arm effect**

| **Outcome** | **Model** | **Difference, percentage points** | **95% CI** | **P value** | **Scenarios improved / worsened** |
| --- | --- | --- | --- | --- | --- |
| Unsafe major error-free | GPT-5.3 | +4.4 | 0.0 to 10.0 | .250 | 3 / 0 |
|  | Gemini 3 Flash | +18.9 | 10.0 to 28.3 | <.001 | 14 / 0 |
|  | DeepSeek V3.2 | +16.1 | 7.2 to 26.7 | .003 | 13 / 4 |
| Exact concordance | GPT-5.3 | +11.7 | 3.3 to 20.6 | .010 | 8 / 2 |
|  | Gemini 3 Flash | +36.1 | 23.9 to 48.9 | <.001 | 23 / 2 |
|  | DeepSeek V3.2 | +16.7 | 7.8 to 26.7 | .001 | 11 / 1 |

**Between-model heterogeneity in the scenario-level difference**

| **Outcome** | **F statistic** | **P value** |
| --- | --- | --- |
| Unsafe major error-free | F(2, 59) = 6.97 | .002 |
| Exact concordance | F(2, 59) = 6.38 | .003 |

*The Firth model does not account for within-scenario clustering and does not replace the cluster-aware generalized estimating equations; it is reported as a sensitivity analysis addressing separation. The direction of effect remained consistent, whereas the very wide profile penalized likelihood confidence intervals confirm substantial effect-size instability associated with sparse and zero-event cells. For the scenario-level analysis the three repetitions for each scenario, model and arm were averaged; confidence intervals were obtained by resampling scenarios with replacement. P values were obtained by exact sign-flip permutation enumeration where computationally feasible, that is by evaluating every sign pattern of the non-zero scenario differences; for the Gemini 3 Flash exact-concordance comparison, in which 25 scenarios differed between arms, a Monte Carlo sign-flip permutation test with 200 000 draws was used instead. For GPT-5.3 and the unsafe major error-free outcome only three scenarios differed between arms, so the smallest attainable P value is .250 and the reported value lies at that floor. Between-model heterogeneity was tested by a cluster-robust Wald F test from an ordinary least squares model of the scenario-level difference on model, with standard errors clustered by scenario.*
