# Supplementary material for: Safety-Oriented Benchmarking of Large Language Models in Risk-Based Management of Abnormal Cervical Screening Results: Scenario-Based Benchmark Study
Source: J Med Internet Res. 2026 Sep 22;28:e98131. doi: 10.2196/98131 (PMC13643256; doi:10.2196/98131)
Supplement: Supplementary file 5 [file jmir_v28i1e98131_app7.docx]

**Multimedia Appendix 7**

**Completed CHART checklist**

*Safety-Oriented Benchmarking of Large Language Models in Initial ASCCP Risk-Based Management of Abnormal Cervical Screening Results: Scenario-Based Benchmark Study*

*Reporting guideline: CHART Collaborative. Reporting guideline for chatbot health advice studies: the CHART statement. Artif Intell Med. 2025;168:103222.*

*Section names refer to the revised manuscript. Where an item is not applicable to this study design, this is stated together with the reason.*

| **Section** | **Item** | **CHART reporting item** | **Where reported** |
| --- | --- | --- | --- |
| **TITLE AND ABSTRACT** | | | |
| Title | **1a** | State that the study is assessing one or more generative AI-driven chatbots for clinical evidence or health advice. | Title. The title identifies the study as a scenario-based benchmark of large language models in ASCCP risk-based management. |
| Abstract | **1b** | Apply a structured format, if applicable. | Abstract. Structured under Background, Objective, Methods, Results and Conclusions. |
| **INTRODUCTION** | | | |
| Background | **2a** | State the scientific background, rationale, and healthcare context, referencing relevant literature. | Introduction, paragraphs 1 to 4. Covers the ASCCP risk-based framework, its complexity, and prior evaluations of large language models in clinical and cervical-screening contexts. |
|  | **2b** | State the aims and research questions including the target audience, intervention, comparator(s), and outcome(s). | Introduction, final paragraph, and Abstract Objective. Three models are compared under two prompt conditions, with the unsafe major error-free rate as the primary outcome. |
| **METHODS** | | | |
| Model identifiers | **3a** | State the name and version identifier(s) of the models and chatbots, and their date of release or last update. | Methods, Models Evaluated. Model names, versions and the testing window (25-31 March 2026) are reported. Query-level build identifiers were not retained; this is stated in the Limitations. |
|  | **3b** | State whether the models and chatbots are open-source or closed-source or proprietary. | Methods, Models Evaluated. All three were accessed as proprietary consumer web interfaces. |
| Model details | **4a** | State whether the model was a base model or a novel base, tuned, or fine-tuned model. | Methods, Models Evaluated. Publicly released base models were used without tuning or fine-tuning. |
|  | **4b** | If a base model is used, cite its development in sufficient detail to identify the model. | Methods, Models Evaluated. Provider and version are given. Development details are not publicly documented for these proprietary systems; this constraint is described in the Limitations. |
|  | **4c** | If a novel base, tuned, or fine-tuned model is used, describe the pre- and post-deployment data and parameters. | Not applicable. No model was tuned or fine-tuned for this study. |
| Prompt engineering | **5a** | Describe the evolution of study prompt development. | Methods, Prompt Design. Two prompt conditions were prespecified before data collection: a baseline clinical prompt and a guideline-directed prompt package. |
|  | **5ai** | Describe the sources of prompts. | Methods, Prompt Design. Prompts were constructed by the investigators from the ASCCP decision framework. |
|  | **5aii** | State the number and characteristics of the individuals involved in prompt engineering. | Methods, Prompt Design and Evaluation Process. Both prompt conditions were developed by the two investigators, who are obstetrics and gynecology specialists. |
|  | **5aiii** | Provide details of any patient and public involvement during prompt engineering. | Not applicable. No patients or members of the public were involved; all scenarios are synthetic. |
|  | **5b** | Provide study prompts. | Multimedia Appendix 1 contains the verbatim prompt templates for all three arms. |
| Query strategy | **6a** | State route of access to the generative AI model. | Methods, Models Evaluated. Access was through the publicly available free web interface of each provider, without an application programming interface and without user accounts. |
|  | **6b** | State the date(s) and location(s) of queries including day, month, year, city and country. | Methods, Repetition and Data Collection. Queries were conducted between 25 and 31 March 2026 in Ankara, Turkiye. |
|  | **6c** | Describe whether prompts were input into separate chat sessions. | Methods, Prompt Design and Repetition and Data Collection. Every query was initiated in an independent, non-account-linked session with no carryover of conversational context. |
|  | **6d** | Provide all generative AI-driven chatbot output and responses. | **NOT MET. The verbatim response texts were not prospectively archived and no recoverable session history was subsequently available. This is reported in the Methods, in the Limitations and in the Data Availability statement. The coded observation-level classifications for all 1080 responses are provided in Multimedia Appendix 8.** |
| Performance evaluation | **7a** | Define the ground truth or reference standard used to define successful performance. | Methods, Gold Standard and Guideline Framework, and Multimedia Appendix 9, which lists the prespecified decision for each of the 60 scenarios with its ASCCP source reference. |
|  | **7b** | Describe the process undertaken for performance evaluation. | Methods, Evaluation Process and Endpoints and Error Classification. A three-tier classification was applied with seven prespecified error-subtype options. |
|  | **7bi** | State the number and characteristics of team members involved in performance evaluation. | Methods, Evaluation Process. Two obstetrics and gynecology specialists scored all responses. |
|  | **7bii** | Provide details of any patient and public involvement during the evaluation process. | Not applicable. No patients or members of the public were involved in evaluation. |
|  | **7biii** | State whether evaluators were blinded to the identity of the chatbots under assessment. | Methods, Evaluation Process. Evaluators were blinded to model and prompt-arm identity but were not independent of gold-standard construction, since both had participated in developing the answer key. This is described in the Limitations. |
| Sample size | **8** | Report how the sample size was determined. | Methods, Sample Size. A precision-based justification was used rather than a conventional power calculation. |
| Data analysis | **9a** | Describe statistical analysis methods, including any evaluation of reproducibility. | Methods, Statistical Analysis. Cluster-adjusted proportions, generalized estimating equations, a Firth penalized sensitivity analysis, a complementary scenario-level paired analysis and inter-rater agreement are described. Intra-model reproducibility across three repetitions is reported in Multimedia Appendix 4. |
| **RESULTS** | | | |
| Results | **10a** | Report the performance evaluation undertaken including alignment between chatbot output and the reference standard. | Results, Primary Endpoint and Exact Concordance sections, with Tables 2 and 3. |
|  | **10b** | For responses deviating from the reference standard, state the nature of the differences. | Results, Error Subtype Distribution, with Table 5. Four error subtypes were assigned among the 165 unsafe major errors. |
|  | **10c** | Report the evaluation for potentially harmful, biased, or misleading responses. | Results, Primary Endpoint and Error Subtype Distribution. The primary outcome is the absence of unsafe major errors, defined as deviations with potential for clinical harm. |
| **DISCUSSION** | | | |
| Discussion | **11a** | Interpret study findings in the context of relevant evidence. | Discussion, Principal Results and Comparison With Prior Work. |
|  | **11b** | Describe the strengths and limitations of the study. | Discussion, Strengths and Limitations. Nine limitations are reported, including evaluator dependence on gold-standard construction, absence of archived response texts, absence of query-level version identifiers, and the enriched case mix. |
|  | **11c** | Describe the potential implications for practice, education, policy, regulation, and research. | Discussion, Comparison With Prior Work and Conclusions. |
| **OPEN SCIENCE** | | | |
| Disclosures | **12a** | Report any relevant conflicts of interest for all authors. | Conflicts of Interest. None declared. |
| Funding | **12b** | Report sources of funding and their role in the conduct and reporting of the study. | Funding. No external funding was received. |
| Ethics | **12c** | Describe the process undertaken for ethical approval. | Methods, Ethical Considerations, and the Ethics statement. An exemption was granted by the Clinical Research Ethics Committee of Ankara Etlik City Hospital before data collection (Reference No 308763928; 18 March 2026). |
|  | **12ci** | Describe the measures taken to safeguard data privacy of patient health information. | Methods, Ethical Considerations. All scenarios are fully synthetic; no patient data, biological material or identifiable health information were used. |
|  | **12cii** | State whether permission or licensing was obtained for the use of original, copyrighted data. | Not applicable. No copyrighted material was reproduced. Clinical scenarios were constructed by the investigators from published guideline decision nodes. |
| Protocol | **12d** | Provide a study protocol. | A separate registered protocol was not published. The prespecified design, endpoints, scoring framework and error-subtype categories are described in the Methods and in Multimedia Appendices 1, 3 and 9. |
| Data availability | **12e** | State where study data, code repository, and model parameters can be accessed. | Data Availability. The coded evaluation dataset (Multimedia Appendix 8), the prespecified gold-standard decisions (Multimedia Appendix 9), the prompt templates (Multimedia Appendix 1) and the analysis code (Multimedia Appendix 10) accompany this submission. The verbatim response texts are not available. Model parameters are not publicly disclosed by the providers. |

**Note on item 6d**

Item 6d is the single checklist item that is not met. All queries were conducted through free, publicly available web interfaces in independent, non-account-linked sessions. No account-linked session history was generated, and the verbatim response texts were not prospectively archived as part of the study dataset; they do not survive in any form. Responses were classified at the time of review against the prespecified reference standard. The material available for independent inspection is therefore the coded classification dataset, together with the gold-standard decisions and the prompt templates. This permits reproduction of every reported count, proportion and test statistic, and inspection of the reference standard, but not independent re-adjudication of the mapping from response text to classification category. This constraint is stated explicitly in the Methods, the Limitations and the Data Availability statement.
