# Supplementary material for: Safety-Oriented Benchmarking of Large Language Models in Risk-Based Management of Abnormal Cervical Screening Results: Scenario-Based Benchmark Study
Source: J Med Internet Res. 2026 Sep 22;28:e98131. doi: 10.2196/98131 (PMC13643256; doi:10.2196/98131)
Supplement: Supplementary file 6 [file jmir_v28i1e98131_app9.docx]

**Multimedia Appendix 9. Intra-Model Response Consistency Across Three Repetitions**

| **Model** | **Prompt Arm** | **3/3 Consistent n (%)** | **2/3 Consistent n (%)** | **Inconsistent n (%)** | **Total Scenarios** |
| --- | --- | --- | --- | --- | --- |
| GPT-5.3 | Baseline Clinical | 59 (98.3%) | 1 (1.7%) | 0 (0.0%) | 60 |
| GPT-5.3 | Structured Guideline | 57 (95.0%) | 3 (5.0%) | 0 (0.0%) | 60 |
| Gemini 3 Flash | Baseline Clinical | 54 (90.0%) | 6 (10.0%) | 0 (0.0%) | 60 |
| Gemini 3 Flash | Structured Guideline | 57 (95.0%) | 3 (5.0%) | 0 (0.0%) | 60 |
| DeepSeek V3.2 | Baseline Clinical | 52 (86.7%) | 8 (13.3%) | 0 (0.0%) | 60 |
| DeepSeek V3.2 | Structured Guideline | 56 (93.3%) | 4 (6.7%) | 0 (0.0%) | 60 |

*Consistency assessed by comparing the consensus classification across three independent repetitions of each scenario. 3/3 = identical classification in all three runs. Across the 360 scenario-model-arm cells, the three repetitions were unanimous in 335 cells (93.1%). The 25 non-unanimous cells are marked individually in Multimedia Appendix 5.*
